# Neonatal administration of synthetic estrogen, diethylstilbestrol to mice up-regulates inflammatory *Cxcl*chemokines located in the 5qE1 region in the vaginal epithelium

**DOI:** 10.1371/journal.pone.0280421

**Published:** 2023-03-16

**Authors:** Ayaka Kitamura, Chen Jiayue, Tomoya Suwa, Yasuhiko Kato, Tadashi Wada, Hajime Watanabe

**Affiliations:** 1 Department of Biotechnology, Osaka University, Suita-Shi, Osaka, Japan; 2 Nucleic Acid Regulation (Yoshindo) Joint Research Laboratory, Graduate School of Engineering, Osaka University, Suita-Shi, Osaka, Japan; University of Salerno, ITALY

## Abstract

A synthetic estrogen, diethylstilbestrol (DES), is known to cause adult vaginal carcinoma by neonatal administration of DES to mice. However, the carcinogenic process remains unclear. By Cap Analysis of Gene Expression method, we found that neonatal DES exposure up-regulated inflammatory *Cxcl* chemokines 2, 3, 5, and 7 located in the 5qE1 region in the vaginal epithelium of mice 70 days after birth. When we examined the gene expressions of these genes much earlier stages, we found that neonatal DES exposure increased these *Cxcl* chemokine genes expression even after 17 days after birth. It implies the DES-mediated persistent activation of inflammatory genes. Intriguingly, we also detected DES-induced non-coding RNAs from a region approximately 100 kb far from the *Cxcl*5 gene. The non-coding RNA up-regulation by DES exposure was confirmed on the 17-day vagina and continued throughout life, which may responsible for the activation of Cxcl chemokines located in the same region, 5qE1. This study shows that neonatal administration of DES to mice causes long-lasting up-regulation of inflammatory *Cxcl* chemokines in the vaginal epithelium. DES-mediated inflammation may be associated with the carcinogenic process.

## Introduction

Endocrine-disrupting chemicals (EDCs) have been known to cause many adverse effects on female reproductive organs and hormone-related cancer progression [1]. Between 1948–1976, diethylstilbestrol (DES), a synthetic nonsteroidal estrogen, was prescribed to pregnant women for the prevention of bleeding, miscarriage, and related complications of pregnancy [2]. In the 1970s, it was found that fetal exposure to DES through their mother during pregnancy enhances the risk of vaginal and cervical clear-cell adenocarcinoma after maturation [3–5]. A mouse model system has been developed to explore the mechanisms of cancer progression [5–8]. These models indicate that neonatal DES exposure in mice induces persistent vaginal epithelial cell proliferation independently of estrogen [9–11]. It seems that DES activates Estrogen Receptor alpha (ERα) through phosphorylation and the continuous phosphorylation of ERα allows cells to accept growth factor signaling [12, 13]. ERα plays an essential role in vaginal development at the early neonatal stage but not at the later stage [14]. Interestingly, stromal ERα but not epithelial ERα is responsible for the proliferation of vaginal epithelium, and the differentiation of epithelium needs epithelial ERα [14]. These findings indicate that the different action of ERα in epithelial and stromal cells is critical for the proliferation control of vaginal epithelial cells. Hormone-related molecules are associated with spatiotemporal regulation in female organs; hence, it implies that neonatal DES exposure may differently affect epithelial and stromal cells during the growing stage.

Chronic inflammation can lead to carcinogenesis since immunity toward tumor growth is uncontrollable [15]. Chronic inflammatory conditions gradually cause dysregulation of immune cells such as T cells and allow cancer cells to grow [16]. Some chemokines such as the C-X-C motif ligand 1 (CXCL1), CXCL2, CXCL3, and CXCL8, play roles in a T cell immune response, and they are up-regulated in human breast cancer [17]. Interestingly, genes encoding these CXCL chemokines locate in the 4q21 region as a gene cluster in humans. The up-regulation of the gene cluster might be related to carcinogenesis by chronic inflammation [17]. Interestingly, this chemokine gene cluster is also conserved in the mouse 5qE1 region.

Recent advances in genome-wide sequence technology have revealed non-coding transcripts which are not translated into protein but have some functions. Long non-coding RNA (lncRNA) and enhancer RNA (eRNA) are being extensively characterized among them [18]. eRNA can interact with both transcription factors (TFs) and mediator complexes, leading to a looping structure within a DNA region between promoter and enhancer [19–22]. Straddling over and combining long distances of DNA are responsible for dynamically-reconstituted and three-dimensional (3D) genomic architecture, resulting in a Super-enhancer structure [19, 23]. Firstly, TFs stimulate transcription from the enhancer region to produce eRNA, and then eRNA can induce the 3D architecture. Indeed, ERα-binding to its ligand estrogen affects eRNA synthesis, resulting in ERα-mediated Super-enhancer structure [22]. In this context, eRNA behaves like a reinforcing bar required to connect enhancer and promoter regions. In contrast, others have shown that transcription reaction itself for producing eRNA plays a significant role in constructing the building blocks [24]. In this context, eRNA is not a simple physical connector between them. Because DES binds to ERα and the complex stimulates transcription from target genes, the ERα-DES complex may induce eRNA synthesis. The eRNA may contribute to the maintenance of the Super-enhancer structure for an extended period regardless of the estrogen. The facts that several cancer-specific Super-enhancers have been identified may suggest the possibility that DES-dependent eRNA synthesis by neonatal DES exposure following might involve vaginal cancer-specific Super-enhancer construction.

In this study, we observed that inflammation-related genes, *Cxcl* chemokines, were prominently and persistently up-regulated by neonatal DES exposure. The expression of *Cxcl* chemokines seems to be related to the non-coding RNA transcribed from regions approximately 100 kb upstream of the *Cxcl* chemokine gene cluster. We also found a different expression pattern of *Cxcl* genes between epithelial and stromal cells.

## Results

### Preparation of a mouse model of vaginal carcinoma

**Fig 1:**
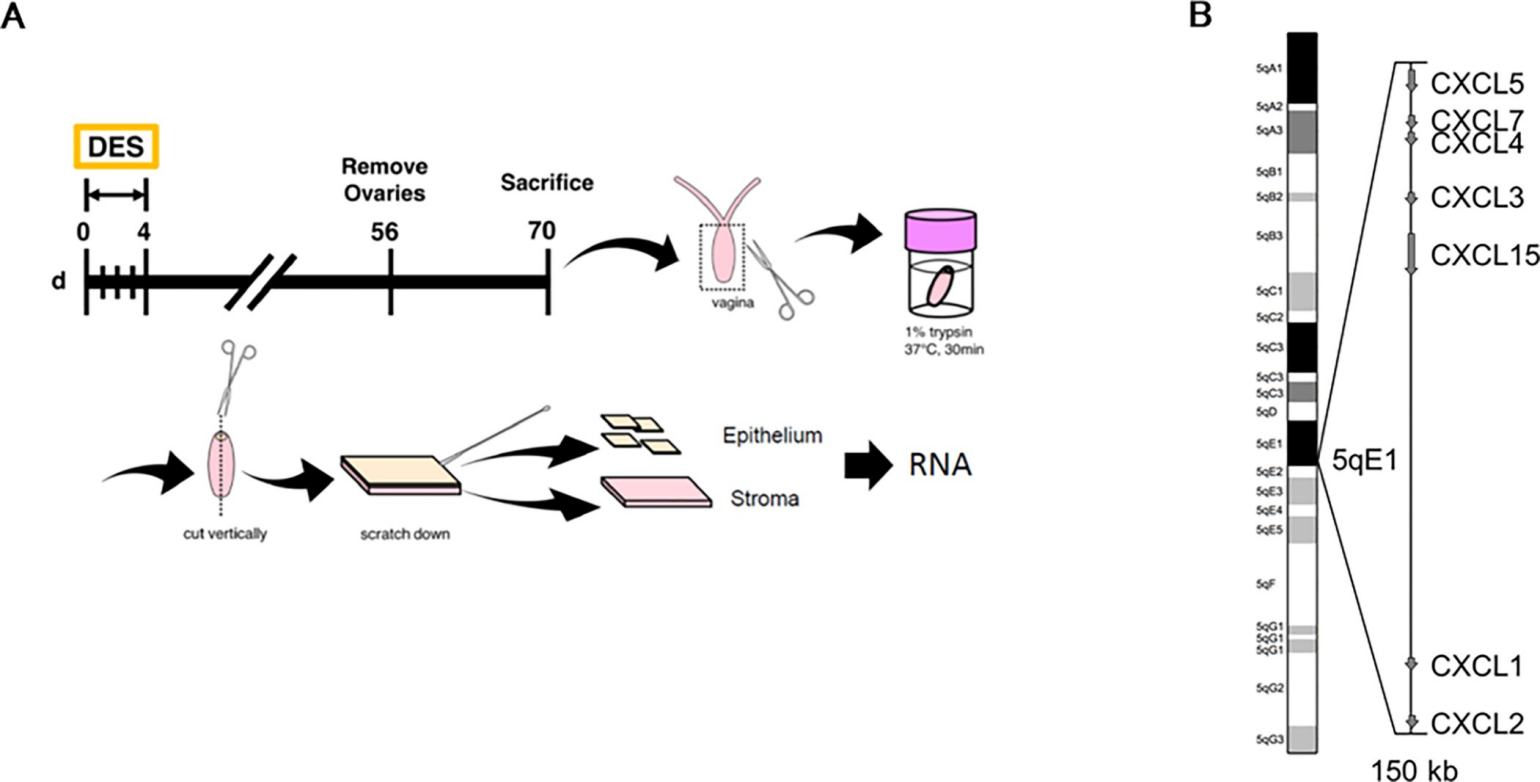
Selection of DES-dependent target genes and *Cxcl* chemokine gene cluster. (A) Workflow shows the sequential steps and criteria to select DES-dependent target genes using DAVID and qRT-PCR. (B) Schematic representation of mouse chromosome 5 showing that multiple *Cxcl* chemokine genes are present in the 5qE1 region of chromosome 5. *Cxcl*1, *Cxcl*2, *Cxcl*3, *Cxcl*4, *Cxcl*5, *Cxcl*7, and *Cxcl*15 belong to the same cluster (150 kb).

Mice were injected with DES or Oil (control group) subcutaneously at neonatal days 0–4, ovariectomized (OVX) at 7-8^th^ weeks, and sacrificed at 9-10^th^ weeks (neoDES or neoOil, respectively)(Fig 1). The histological observations of the vagina was used as one of the references to identify if neonatal DES exposure was effective or not. The vagina of neoDES mouse was swelling due to the proliferation of epithelial cells compared to control group one (neoOil) (S1A Fig). The abnormal cell proliferation also resulted in an increase in vagina weight to some extent. The influence was much more prominent on the amount of extracted total RNA from the vagina (S1B Fig). In addition, neonatal DES exposed mice (neoDES) showed more than 3 cell layers in the epithelium, whereas the epithelium of neoOil was composed of only 1–2 epithelial cell layers (S1C Fig). These observations were consistent with previous many studies, and we used these phenotypes to confirm the effectiveness of DES injection [7].

### DES administration up-regulates *Cxcl* chemokines in the 5qE1 region

**Fig 2:**
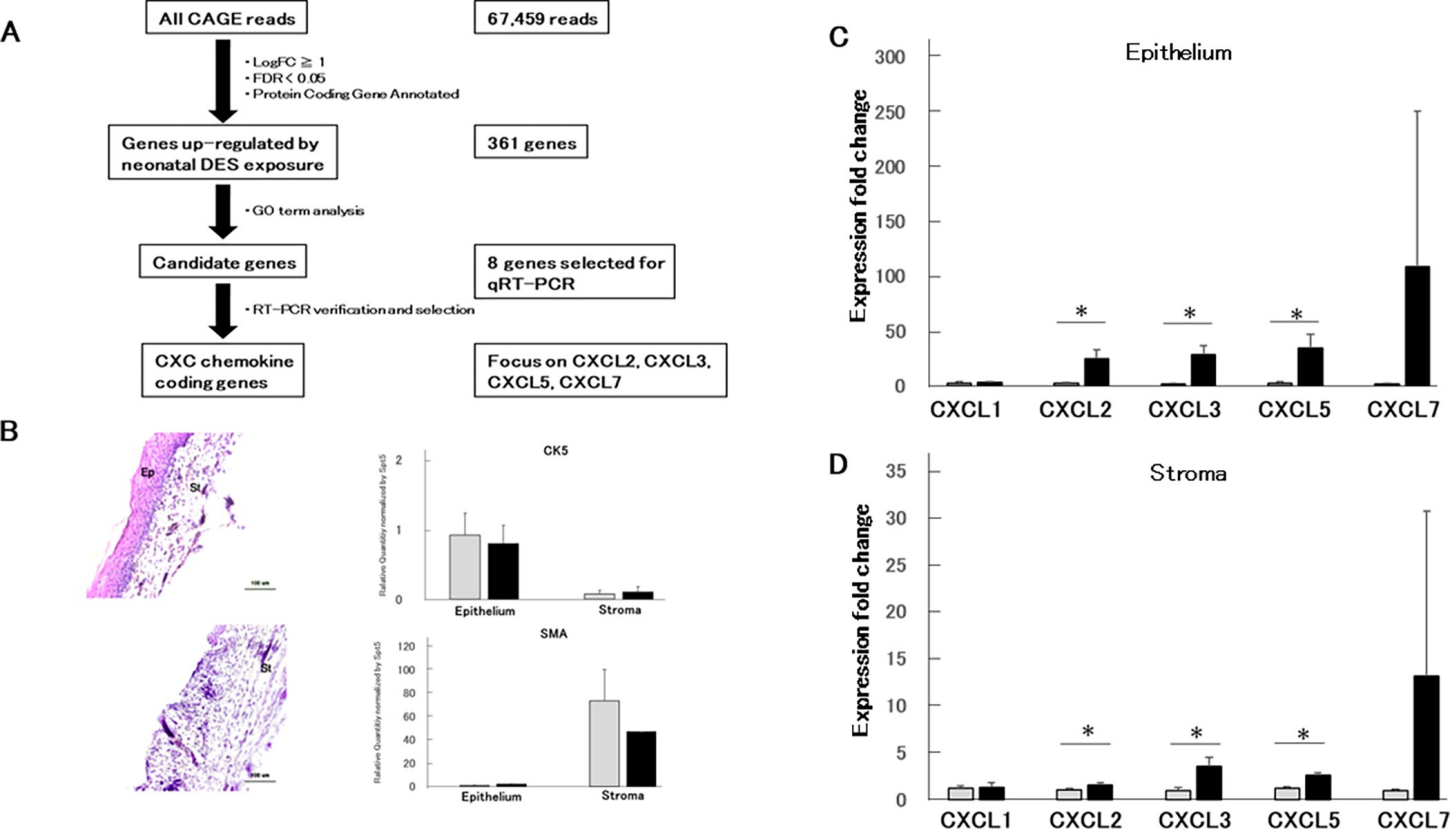
Epithelium-stroma separation method and *Cxcl* chemokine expression. (A) The procedures of tissue separation from the intact vagina are illustrated. See Materials and Methods for more details. OVX and sacrifice were performed at 8^th^ week and 10^th^ week after birth, respectively. (B) The separated epithelium and stroma were subjected to H&E stained (Ep, Vagina Epithelium; St, Vagina Stroma) and total RNA extraction following qPCR analysis. Expression fold changes of CK5 (keratin 5) and SMA (α-smooth muscle actin) genes between epithelium and stroma. Boxes in gray and black indicate neoOil and neoDES, respectively. Expression fold change between neoOil and neoDES in the epithelium (C) or stroma (D). Genes indicated were analyzed (n = 3; t-test, *p < 0.05).

Cap Analysis of Gene Expression (CAGE) is a technology to capture the 5’ end of total RNA and map transcriptional start sites (TSSs) as well as DNA regulatory elements including promoter and enhancer [25]. The technology allows us to compare gene expression levels between DES-exposed and non-exposed samples. All CAGE reads consist of 67459, and we selected 361 reads as genes up-regulated by neonatal DES exposure (Fig 2 and S1 Table). Then, we annotated Gene Ontology (GO) terms on DAVID (The Database for Annotation, Visualization, and Integrated Discovery) of the 361 genes to examine which biological functions are most significantly changed [26] (S1 Table). According to results from these analyses, we finally reached eight candidate genes, and we chose four *Cxcl* chemokine genes, including *Cxcl*2, *Cxcl*3, *Cxcl*5, and *Cxcl*7, based on results of quantitative PCR (qPCR) analysis (Fig 2, Table 1 and S2 Table). These genes are ligands of CXC chemokine receptor 2 (CXCR2) and known to be involved in angiogenesis in a tumor. Interestingly, genes encoding the *Cxcl* chemokines condense in the 5qE1 region, a gene cluster (Fig 1B).

**Table 1:** GO analysis of the CAGE results.

Term	P Value	Genes
GO:0045236 CXCR chemokine receptor binding	9.35 X 10^−6^	CXCL1, PPBP, CXCL5, CXCL3, CXCL2
GO:0043565 sequence-specific DNA binding	3.99 X 10^−5^	EGR1, MAFF, JDP2, FOSL2, RREB1, IRX2, STAT5A, ESRRG, NR4A1, FOSB, GRHL1, JUNB, HES1, FOS, ATF3, DBP, NR1D2, SPDEF, FOXE1, PRDM1, RERE, PITX1, KLF4
GO:0001077 transcriptional activator activity, RNA polymerase II core promoter proximal region sequence-specific binding	2.77 X 10^−4^	ERG1, STAT5A, SOX11, NR4A1, FOSB, GRHL1, JUNB, FOS, DBP, ZFP750, CYS1, KLF4, PITX1
GO:0008009 chemokine activity	4.28 X 10^−4^	CXCL1, PPBP, CXCL5, CXCL3, CXCL2, CCL8
GO:0005125 chemokine activity	6.03 X 10^−4^	CXCL1, LIF, INHBB, CSF3, TNF, CXCL5, CXCL3, IL1RN, CXCL2, CCL8, TGFB1

### Epithelial- stromal separation

Previous results showed that even in the same organ, vagina in this study, phenotype varies between epithelium and stroma [10]. Another study also reported that gene expression patterns were different between epithelium and stroma in the vagina in estradiol exposed mice [11], indicating that the pathogenic mechanism is different between these two tissues. Therefore, tissue-specific analysis is required to study the DES-mediated carcinogenesis mechanism and practical method to isolate total RNA from separated vaginal epithelium and stroma needs to be established.

As shown in Fig 1, the epithelial-stromal separation method was re-designed in accordance with the methods described previously [9, 11, 27] (see Materials and Methods for more information). Total RNA was extracted and purified from separated tissues and then subjected to Bioanalyzer™ to check the quality of total RNA. According to the previous study [28], an RNA integrity number (RIN) value larger than 5 is desirable for a reliable qPCR result in general. S2A Fig showed that most of the total RNA samples prepared by our methods had RIN values large enough for qPCR to produce reliable data. Total RNA from epithelial samples showed high quality, with most of their RIN value larger than 8. Separated epithelium and stroma were also confirmed histologically. We made tissue sections and Hematoxylin & eosin stained. Fig 2B and S2B–S2E Fig showed the condition of the vagina before and after epithelium stripping. Most of the epithelial cells were eliminated from the stroma (S2C Fig). We then investigated whether the extracted total RNA from separated epithelium and stroma keeps their genetic characteristics using epithelium marker Keratin 5 (CK5) [29, 30] and stroma marker Alpha-smooth muscle actin (SMA) [29]. As shown in Fig 2B, the SMA expression level was remarkably decreased in epithelial samples, indicating the purity of isolated epithelium. Also, the CK5 expression level was significantly reduced in stromal sections, again suggesting the success in the separation of epithelium.

### The induction level of *Cxcl* chemokines in epithelial cells is higher than that in stromal cells

Using neoDES mouse and neoOil mouse at day 70, we examined the expression levels of the *Cxcl* chemokine genes in epithelium and stroma by qPCR. In epithelium (Fig 2C), *Cxcl*2, *Cxcl*3, *Cxcl*5, and *Cxcl*7 expression were highly up-regulated, while *Cxcl*1 was not. These results were consistent with the, expression level expected from CAGE data (S2 Table). In stroma (Fig 2D), we observed up-regulation of *Cxcl*2, *Cxcl*3, *Cxcl*5, and *Cxcl*7 genes, but the expression fold changes were much smaller than those in the epithelium. The expression of *Cxcl*1 was unchanged in the stroma (Fig 2D). In the case of *Cxcl*7, the average expression level of neoDES is much higher than neoOil, but it was statistically insignificant. (Fig 2C and 2D). It may be due to the variability among individuals (see discussion). These results suggest that the up-regulation of Cxcl genes was mainly caused in the epithelium.

### Persistent effect of DES on the expression of the chemokine genes

**Fig 3:**
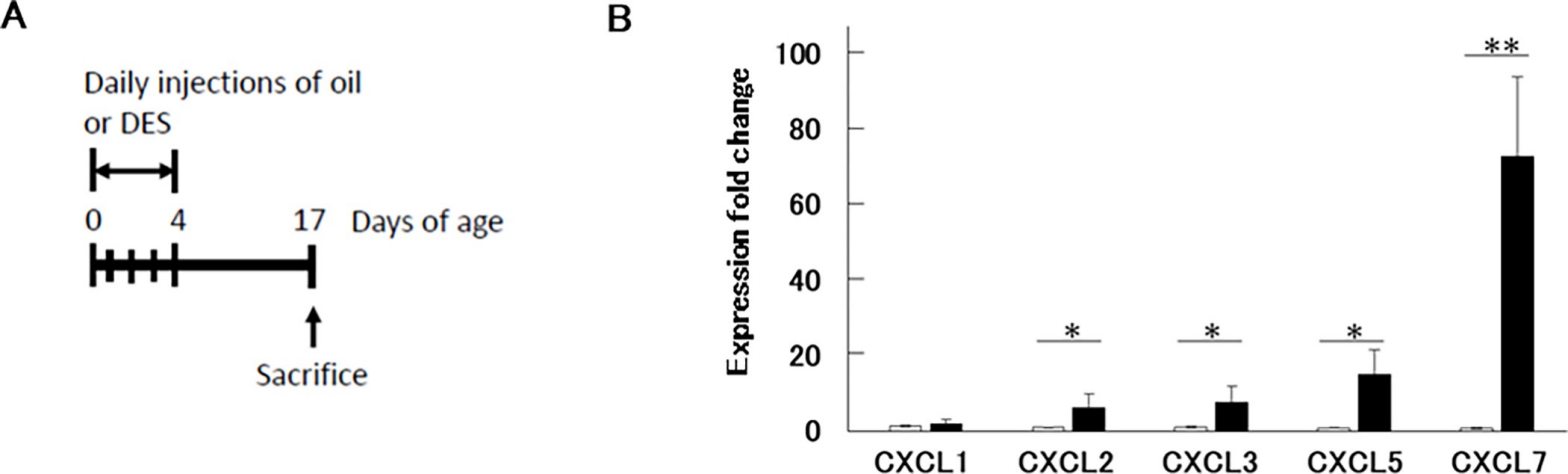
DES-induced expression changes of CXC chemokine genes in day-17 mice vagina. (A) The procedures are illustrated. (B) Total RNA was extracted from the whole vagina and subjected to qPCR analysis. Expression fold change between day-17 neoOil (gray) or neoDES (black) was shown. Genes indicated were analyzed (n = 3; t-test, *p < 0.05, **p < 0.01).

Reproductive organs are controlled by internal hormones during development [31, 32]. Many studies analyzing tumors and cancer cell lines derived from reproductive organs have been conducted and reported that many cancers have some positive correlations with these hormones.[32–34]. However, the activation of the Cxcl genes in this study was observed in ovariectomized mouse, which was neonatally DES exposed. Thus it was possible that the activation of the Cxcl genes was evoked soon after the neonatal DES exposure. To investigate this possibility, we examined the gene expression change much earlier stage. To minimize the influence of internal hormone on our analysis, we collected the vaginas on day-17, before the sexsual maturation (Fig 3A). It was impossible to separate epithelium and stroma and extract RNA by the same method mentioned above because the vagina at day-17 was too small. Therefore, we extracted the total RNA from the whole vagina and performed qPCR. The chemokine genes up-regulated similarly to that of day-70 (Figs 2C and 3B). This result indicates that the expression of inflammation-related genes was chronic and maintained for more than six weeks from day-17 to day-70. The result also suggests that the internal estrus stimuli after sexual maturation may not be involved in the long-lasting inflammation in the vagina.

### DES administration increased in non-coding transcripts derived from about 100 kb far from the gene cluster of *Cxcl* chemokines

Super-enhancer is known to rearrange the chromosome structure through protein-nucleotides interaction, including transcription factors, mediators, RNA polymerase II, DNA, and eRNA [35]. eRNA can regulate gene expression from the promoter in a *cis* and *trans* manner [36], leading to a long-lasting change of genome-wide transcriptomes [35]. Our finding that the Cxcl genes are activated for a long period rised the possibility that eRNA controls the expression of the Cxcl genes

Because eRNAs are consistently found within 200 kb from the promoter region of its target gene, we investigated non-coding transcripts within 200 kb upstream and downstream of the gene cluster of *Cxcl* genes located on the same chromosome [37]. To find enhancer regions transcribed into eRNAs by neonatal DES exposure, the CAGE data was integrated with publicly available sequencing data in UCSC Genome Browser [38]. As a result, two putative eRNAs were found 100 kb upstream of the cluster of CXC chemokine coding genes (S3A Fig), defined as peRNA1 and peRNA2 in this study (S3B Fig). H3K27 acetylation and H3K4 mono-methylation are well recognized as a marker for active enhancers, and indeed their marks around the eRNA1 and eRNA2 regions are observed in placenta samples (S3B Fig).

**Fig 4:**
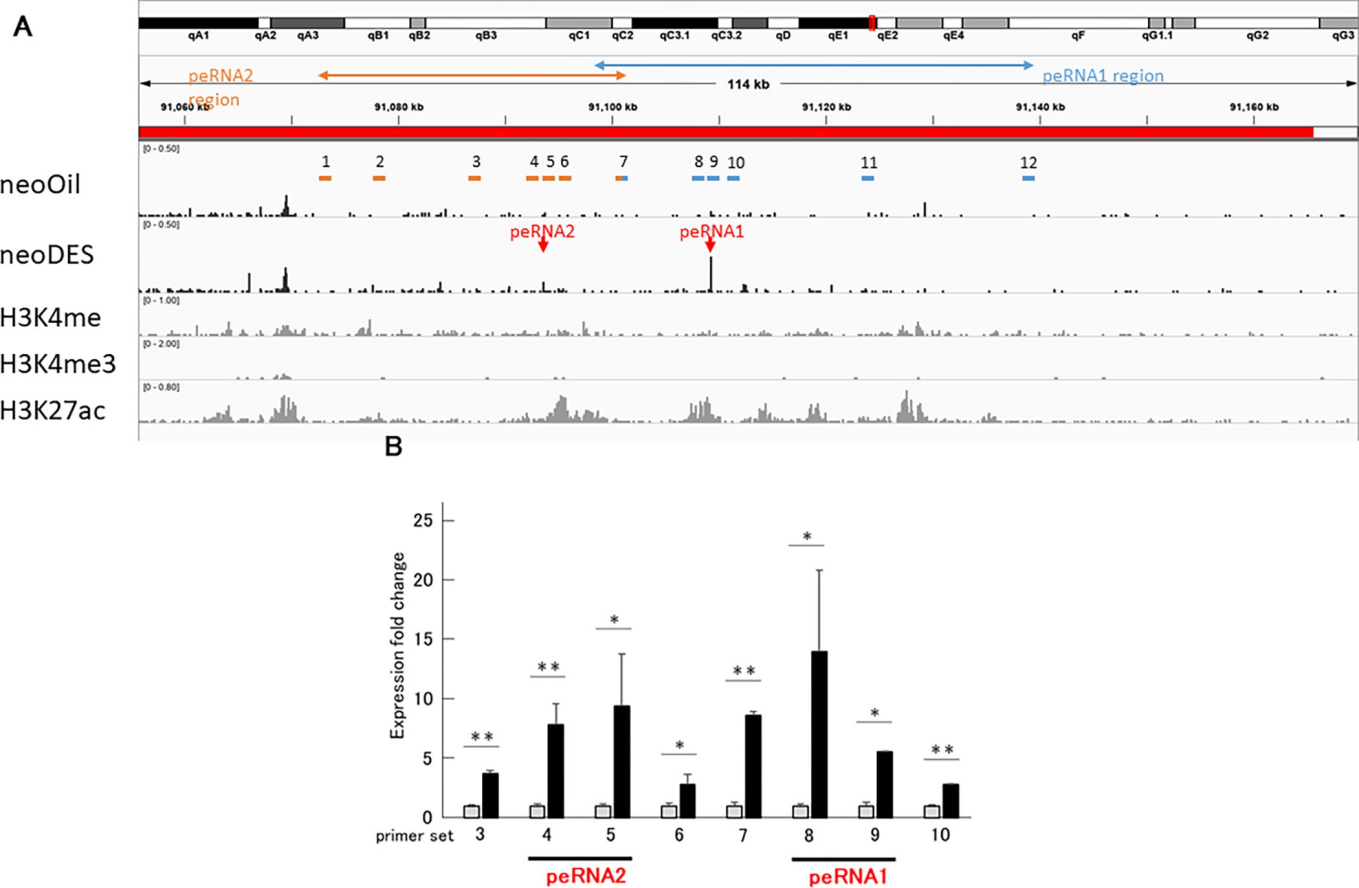
DES-induced changes in the expression of putative eRNAs in the vaginal epithelium. (A) IGV Screen Shot of CAGE results showing designed 12 primer sets. The location of the primer sets is indicated. Two arrows in red show peaks of CAGE reads we detected in neoDES but not in neoOil. Information on placenta histone modification patterns, including H3K4m1, H3K4m3, and H3K27a are shown. See S3 Fig for more details. (B) Expression fold change between neoOil (gray) and neoDES (black) in the epithelium. Results of qPCR analysis with primer sets indicated are shown (n = 3; t-test, *p < 0.05, **p <0.01). Peaks of CAGE reads corresponding to peRNA1 and peRNA2 are present near the primer sets indicated with horizontal lines, respectively.

Then we examined the expression of these putative eRNAs by qPCR using the RNA obtained from epithelial cells. Twelve primer sets were designed as shown in Fig 4A. Primer sets No. 4, 5, 6, and 8, 9, 10 were designed within 1 kb up/downstream of the Transcription Start Sites (TSSs) to detect the peRNAs (Fig 4A). Primer sets No. 1, 2, 11, and 12 were designed more than 10 kb away from the TSS of peRNA1 and peRNA2, as negative controls (Fig 4A). Primer sets No.3 and 7 were located between the TSS and negative control primers (Fig 4A) and the detailed information about primer sets is listed in S3 Table. We tested the 12 primer sets, and the foldchanges of peRNAs expression by neonatal DES exposure was evaluated. As shown in Fig 4B, up-regulation of peRNA1 and peRNA2 by neonatal DES exposure was confirmed. By neonatal DES exposure, the expression of peRNA1 and peRNA2 were activated about 12-fold and 8-fold, respectively (Fig 4B).

### peRNA1 and peRNA2 up-regulated from early stage

**Fig 5:**
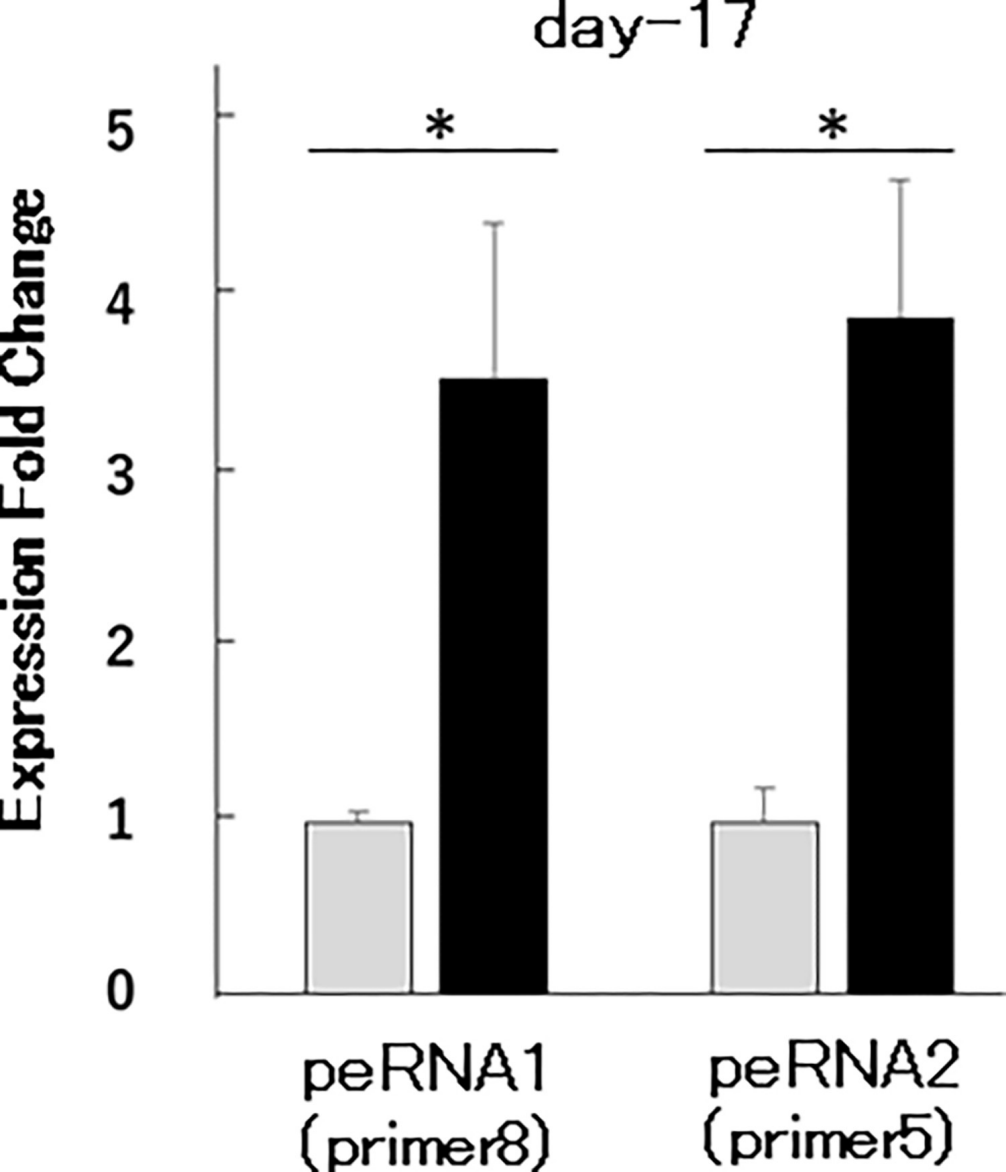
Expression pattern of peRNA1 and peRNA2 in mice vagina at day-17. Expression fold change of peRNA1(primer 8) and peRNA2 (primer 5) between neoOil (gray) and neoDES (black) (n = 3; t-test, *p < 0.05).

As our finding prove that both the Cxcl genes and the peRNAs are activated in adult mouse after neonatal DES exposure, then we examined if the peRNA expression was also activated in early stage (day-17). We chose No.8 as a primer for peRNA1 and No.5 as a primer for peRNA2. Total RNA extracted from day-17 whole vagina was subjected to qPCR analysis with primer sets No. 5 and No. 8. As shown in Fig 5, the up-regulation of peRNA1 and peRNA2 in the early-stage (day-17) was confirmed.

## Discussion

In this study, we showed for the first time that administration of DES to mice in the neonatal period causes persistent up-regulation of inflammatory *Cxcl* chemokines in the vaginal epithelium (Fig 2). In addition, we present a possible involvement of two putative eRNAs in this up-regulation (Figs 4 and 5). The chemokine genes exist in a gene cluster and the peRNAs production occurs nearby the cluster region. The persistent up-regulation of inflammatory chemokines possibly caused by peRNA expression may lead to chronic inflammatory conditions, resulting in vaginal cancer. In humans, a series of CXC chemokine genes locate in a short-range (360 kb) of the long arm of chromosome 4, and their overexpression seems to be associated with breast cancer [17]. These indicate that revealing how peRNAs controls CXC chemokine gene transcription can clarify carcinogenic mechanisms in humans.

When we analyzed total RNA collected from the day-17 vagina, DES administration to neonatal mice showed the strong induction of the *Cxcl*7 gene, which was more than 70-fold induction (p < 0.001, Fig 3B). We observed similar strong induction in the epithelium sample at day-70 vagina though its p-value was more than 0.05 (Fig 2C). It may be possible that the abnormal inflammatory condition was caused by the high expression of *Cxcl*7 or other chemokines in neoDES mice. Since DES-mediated vaginal carcinogenesis occurrence was not 100%, we guess the adaptation to DES response is varied depending on each individual.

Previous studies showed that neonatal DES exposure could induce estrogen-independent activation of several genes involving cell proliferation and differentiation [5, 8]. They indicate that DES exposure disrupts the estrogen-signaling pathway, resulting in estrogen-independent persistent proliferation and cornification of female reproductive organs [5, 8]. However, how DES disturbs these pathways and how the effect is sustained for an extended period after birth remain unclear. One of the hypotheses is that another factor, rather than estrogen, was produced in dependence on neonatal exposure. The produced factor itself or the production process can be continuing till adult mice trigger the carcinogenesis.

Our finding that the peRNA1 and peRNA2 are activated by neonatal DES exposure suggests a possibility that eRNA is the factor that transform the DES exposure memory at neonatal stage (Fig 4). It can be assumed that DES affects the eRNA transcription in the neonatal period with epigenetic modifications resulting in active eRNA transcription continuously. These characteristics were consistent with up-regulated *Cxcl* chemokine genes, suggestin the cis-regulatory link between the two peRNAs and the downstream *Cxcl* chemokine cluster. However, further experiments, such as knocking down the peRNAs to see if *Cxcl* chemokines are down-regulated or not, analyze the binding motif of the enhancer regions are required to confirm their cis-regulatory relationship.

Instead, it may be also possible that DES causes epigenetic modifications, such as H3K4 mono-methylation and H3K27 acetylation, to induce the transcription of eRNAs, which are initially not transcribed in non-treated mice. This modification itself sustains the adult stage, and the up-regulated downstream genes via the transcribed eRNAs will eventually lead to adenocarcinoma in mature reproductive organs. This assumption requires more epigenetic analysis, including histone binding condition and enhancer-binding motif.

Previous studies showed that proliferation and cornification were epithelium-specific [10]. So, due to the different phenotype and behavior between epithelium and stroma in the vagina, epithelium- or stroma-specific analysis based on an epithelium-stroma separation method is necessary. Therefore, we aimed to identify the gene whose expression altered by neonatal DES-exposure and showed different expression patterns in epithelium and stroma of the vagina (Fig 2). Meanwhile, limitation still exists, and improvements in some procedure may lead to a better result. For example, in the RNA quality test, the samples from epithelium showed high enough RIN values, but the RIN values of stroma samples were relatively low (S2A Fig). We can consider some reasons. When scratching down the epithelium from the stroma, the spatula may damage the stroma due to the repetition of scratching. The thicker layer of the stroma may also inhibit Sepasol containing phenol from permeate thoroughly into the tissue. Besides, compared to the high purity of isolated epithelium, there were still a few epithelial cells remaining on the stroma layer (Fig 2B and S2B Fig). Therefore, the fractional contaminant of epithelium in stroma samples needs to be considered when analyzing the expression pattern in the following experiments. It may be also useful to apply the one-cell sequencing for further analysis.

In conclusion, although further investigation is needed, we identified putative eRNA expression in vagina epithelial cell, which is activated by neonatal exposure of DES. In the same region, the Cxcl gene cluster was located and some of the genes showed similar behaviors with putative eRNA. These findings rise the possibility that, by the expression of the eRNA, the expression of Cxcl genes may be activated and stabilized by forming stable complex. Then continuous expression of these cytokines may cause the propagation of the cancer cells.

## Materials and methods

### Animals

Pregnant female Jc1:ICR mice (CLEA, Japan) and Slc: ICR (Japan SLC, Japan) were purchased. The mice were housed under conventional conditions, namely, a constant temperature of 23±2°C and 60±5% humidity with a regular 12 h light and dark cycle. Standard chow (CE-2, Clea Japan) and water were freely available [39]. Generally, three mice were used for each experiment. The mice were anesthetized using the anesthesia with midazolam, medetomidine, and butorphanol tartrate (MMB) combination, The mice were euthanized by manual cervical dislocation by trained personnel manual cervical dislocation under anesthesia. All experiments were conducted under the 2006 Japanese Association for Laboratory Animal Science guidelines for the care and use of experimental animals [39]. The experimental protocols were also approved by the Animal Experiment Committee of Osaka University (Approval No. 25-1-1).

### Model mice

Model mice were prepared under the method described in the previous study^7^. Neonatal mice were given daily subcutaneous injections of 30–80 μL of 0.1 mg/mL DES (DO526, TCI, Japan) solution to administer DES of 3 μg/g or 3μg/pup, or corn oil alone (032–17016, Wako, Japan) for the control group, from day 0 (the day of birth) to day-4. Ovariectomy (OVX) was conducted at day 56 with anesthesia treatment to exclude the effect of endogenous estrogen after the adolescence. The treated mice were sacrificed under anesthetization. We performed the OVX on day 17 before the sexual maturation by cervical dislocation, and then we dissected the vaginas.

### Epithelial- stromal separation

The epithelial-stromal separation method was re-designed in accordance with the methods described in the previous studies (9, 11, 27). The dissected vagina was digested in 1 mL of 1% trypsin (1:250 powder, 9002-07-7, Gibco, Canada) in Hanks’ balanced salt solution (H6648, SIGMA, US), in a 37°C water bath, for 30 min. After digestion, the vagina was placed into cold Hanks’ balanced salt solution to stop the trypsin action. In cold Hanks’ balanced salt solution, the tube-shaped vagina was cut vertically into a sheet shape with dissection scissors. The vaginal epithelium located at the inner side of the tube-shaped organ was scratched down from the stroma by a flat stick, such as the flat side of a spatula. The separated epithelium and stroma were transferred into tubes with Hanks’ balanced salt solution and collected by centrifugation (3,000G, 1 min, 4°C). Remove the Hanks’ balanced salt solution, add 750 μL of Sepasol (Sepasol-RNA I Super G, NACALAI TESQUE, Japan) for each tube, and mix it vigorously to disperse the cells in Sepasol. The epithelium and stroma were homogenized in Sepasol by Micro Smash™ (MS-100, TOMY, Japan) with 4 pieces of 3.0φ beads (ZB-30, TOMY, Japan), 3 times, each time 10 sec, 3,000 rpm. These procedures were conducted to make sure that Sepasol penetrated the cells to avoid RNA degradation. Then the samples can be stored at -30°C.

### Tissue fixation and paraffin embedding

The tissue sample was washed in Hanks’ balanced salt solution and incubated in 4°C, 4% paraformaldehyde phosphate buffer solution (163–20145, Wako, Japan) for 2 hours. The pieces were then washed in 1 X PBS (Phosphate Buffered Saline, 27575–31, NACALAI TESQUE, Japan), and ethanol replacement was carried out for dehydration. First, the pieces were immersed in 70% ethanol overnight. Next, we absorb the pieces in 99.5% ethanol for 30 min, 5 times. Then the pieces were immersed in 100% ethanol (prepared from 99.5% ethanol with molecular sieve (133–08645, Wako, Japan) in advance) for another 30 min. To make paraffin (26142–21, NACALAI TESQUE, Japan) fully permeate into the tissue, we replaced ethanol with Lemosol (128–03993, Wako, Japan) by immersing the sample in Lemosol for 1 hour, 3 times, each time changing to newly prepared Lemosol. The pieces were immersed in 68°C liquid paraffin overnight, followed by another 68°C liquid paraffin, 1 hour, two times, each time changing to newly prepared paraffin. In the end, the tissue was placed in a rectangular container made of aluminum foil in a specific orientation, and pour the liquid paraffin into it. Leave it at room temperature till the paraffin coagulate into solid form and attach the paraffin block to a wooden block (2-173-03, AS ONE, Japan) with melt paraffin.

### Sectioning and hematoxylin & eosin stain

We cut the sample block into 6 μm slices with a microtome (PR-50, Yamato Kohki Industrial, Japan). The sliced samples were extended on APS-coated slide glasses (S8445, Matsunami Glass Ind, Japan), pre-heated with water on them, on a 42°C slide warmer (PS-53, Sakura Seiki, Japan). After the slides were dried, Hematoxylin & eosin stain was conducted.

### Equipment and settings

The sections were observed under a brightfield microscope (OLYMPUS BX50; Olympus) equipped with a CCD camera (KEYENCE VB-7010; KEYENCE).

### Total RNA extraction and purification

In order to recover total RNA from mouse vaginal epithelium and stroma, 200 μL of chloroform (08402–55, NACALAI TESQUE, Japan) was added to each sample tube, mixed by inverting, and incubated at room temperature for 5 min. After incubation, centrifuge the samples (15,000 rpm, 15 min, 4°C). The upper aqueous phase was transferred into new tubes, and 500 μL of 2-propanol (29113–95, NACALAI TESQUE, Japan) was added into it, mixed by inverting, and incubated at room temperature for 15 min. After incubation, centrifuge the samples (15,000 rpm, 15 min, 4°C). The RNA was precipitated at the bottom of the tube, so remove the supernatant from the tube. Then 500 μL of cold 75% ethanol (adjusted from 99.5% ethanol (14713–95, NACALAI TESQUE, Japan)) was added to the pellet for ethanol precipitation. Centrifuge the samples (15,000 rpm, 10 min, 4°C). Remove the supernatant and dry it in the air to eliminate the remaining ethanol. Dissolve the RNA pellet in Ribonuclease-free water and incubate at 60°C for 1 min. Store the total RNA samples at -80°C. The total RNA was later purified with an RNA purification kit (RNA Clean & Concentrator™-5, Zymo Research, US).

### Total RNA analysis

The quantity and quality of total RNA were measured by NanoDrop™ (NanoDrop 2000 Spectrophotometers, Thermo Scientific, US) and Bioanalyzer™ (Agilent2100, Agilent technologies, US).

### Quantitative RT-PCR

cDNA samples were synthesized by reverse transcription PCR from mouse vaginal or epithelial or stromal total RNA by the following procedure. Total RNA (50–300 ng) was reverse-transcribed to the first-strand cDNA using Superscript III Reverse Transcriptase (Invitrogen) and oligo dT primer to the method in the manual. RT-qPCR was then performed by placing 5 μL of a 1:~20 dilution of the cDNA into a well that contained 15 μL of reaction mix: the reaction mix included 0.04 μL of 100 μM primer sets, 0.4 μL of Rox High reagent, and 10 μL of KAPA SYBR Fast qPCR Kit (Nippon Genetics). Reactions were run in at least duplicate on a StepOnePlus real-time PCR machine (Life Technologies) with the following protocol: 95°C (30 s) followed by 40 cycles of 95°C (5 s) and 60°C (30 s). Data were normalized according to the amount of Spt5 using the ΔΔCt method. Primer sequences are listed in S3 Table.

### CAGE-seq and Gene Ontology (GO) analysis

Initial picked read counts were 67,459 reads, including non-coding transcripts reported from DNAFORM. Then we collected the genes indicating more than two-fold change (LogFC ≧1) and under 0.05 of false discovery rate (FDR < 0.05), excluded the repetitive counted genes, and finally curated only the 361 coding genes.

### Gene ontology (GO) analysis

Functional enrichment analyses were performed using the tool Database for Annotation, Visualization, and Integrated Discovery (DAVID) v.6.8; defaults setting were used. The whole mouse genome was used as background. Up-regulated genes filtered from all CAGE reads were analyzed by DAVID and listed according to the three ontology domains (cellular component, molecular function, biological process) and the KEGG pathway. From each list, terms or pathways showing a closer relationship with this adenocarcinoma were picked out. The genes belonging to the selected terms and pathways were listed, and the genes exhibiting crossover among different groups were picked out as candidate genes.

### Integrated analysis

CAGE results were aligned to the mouse reference genome (NCBI37/mm9) using UCSC Genome Browser (http://genome.ucsc.edu/index.html), defaults setting was used. Within the Browser, the data were integrated to UCSC Genes, Ensembl Genes, Histone Mods by ChIP-seq from ENCODE/LICR (LICR Histone (Placenta: H3K4m1, H3K4m3, H3K27a)), to identify the non-coding RNA peaks and enhancer regions. Since H3K4me1, H3K27a, along with the absence of H3K4me3, is considered the criteria of enhancer region (40), up-regulated reads without coding gene annotation and satisfy the criteria mentioned above were defined as putative enhancer RNAs in this study.

### ChIP-seq data processing

The public data were downloaded from the NCBI Gene Expression Omnibus (GSE29184). The single-end reads for histone marks (H3K4me, H3K4me3, and H3K27ac) were obtained and aligned to the mouse genome (mm9; Genome Reference Consortium Mouse Build 37 from July 2007) using Bowtie2 version 2.4.5. The duplicated reads were discarded from the reads using rmdup command of samtools version 1.10. The bigwig files were displayed on the integrative genomics viewer (IGV).

### Statistics

For significance testing, an unpaired t-test was performed with Excel 2017 (Microsoft, Redmond, WA). The value of n that represents the number of independent replicates is described in the Figure legends. *p < 0.05 and **p <0.01 by Student’s t-test. Error bars represent SEM.

## Supporting information

S1 Fig neoOil and neoDES mice. (A) Picture of dissected vagina with uterus. The vagina of control group mice (neoOil) and DES-treated (neoDES). (B) (Left) Dissected vagina weight (neoOil vs neoDES). (n = 10; t-test, *p < 0.05). (Right) Extracted RNA amount from intact vagina (neoOil vs neoDES). (n = 3; t-test, *p < 0.05). As the standard deviation of their body weight was much smaller than the vagina weight, these values were directly compared. (C) Histological observation was performed for the vaginas tissue sections of neoOil and neoDES stained by hematoxylin and eosin. Magnification, ×200; scale bar = 100μm.
(TIF)

S2 Fig Separation of epithelium from the stroma. (A) Electrophoresis result and corresponding RNA Integrity Number (RIN) value provided by Bioanalyzer™. Part of the sample results is shown here. The left 8 samples were from epithelial RNA, and the right 8 samples are from stroma RNA. The upper band in the electrophoresis gel picture shows the 28 S rRNA, and the lower band shows 18 S rRNA. The RIN value is calculated by a set of algorithms using the Agilent Bioanalyzer machine. RIN value varies from 1 to 10, with 1 being the most degraded profile and 10 being the most intact. A Higher RIN value is shown in darker green color in this Figure. (B) to (E) Vaginal histology before and after epithelium-stroma separation. Sections were stained with Hematoxylin and Eosin.
(TIF)

S3 Fig IGV screenShot. (A) CAGE result showing the relationship of CXC chemokine cluster and peRNAs. CAGE result (Control group and neoDES mice) and public ChIP-seq histone track (Placenta H3K4me, Placenta H3K4me3, and Placenta H3K27ac, showing a comprehensive survey of cis-regulatory elements in the mouse genome by using ChIP-seq to identify transcription factor binding sites and chromatin modification profiles in 8th-week mouse (C57BL/6) placenta.) The region including putative eRNAs (a) is about 100 kb upstream of the CXC chemokine cluster region (b). Magnification of (a) and (b) are shown in Figure. (B) The region including peRNA1 and peRNA2. peRNA1 and peNRA2 were up-regulated in neoDES mice. S1SI_Caption>
(TIF)

S1 Table 361 up-regulated transcrits with coding gene annotation. (DOCX)

S2 Table Up-regulated transcripts from genes related to *Cxel* chemokine. (DOCX)

S3 Table Primer list. (DOCX)
