# Supplementary figures and images for: Neonatal administration of synthetic estrogen, diethylstilbestrol to mice up-regulates inflammatory Cxclchemokines located in the 5qE1 region in the vaginal epithelium
Source: PLoS One. 2023 Mar 16;18(3):e0280421. doi: 10.1371/journal.pone.0280421 (PMC10019738; doi:10.1371/journal.pone.0280421)

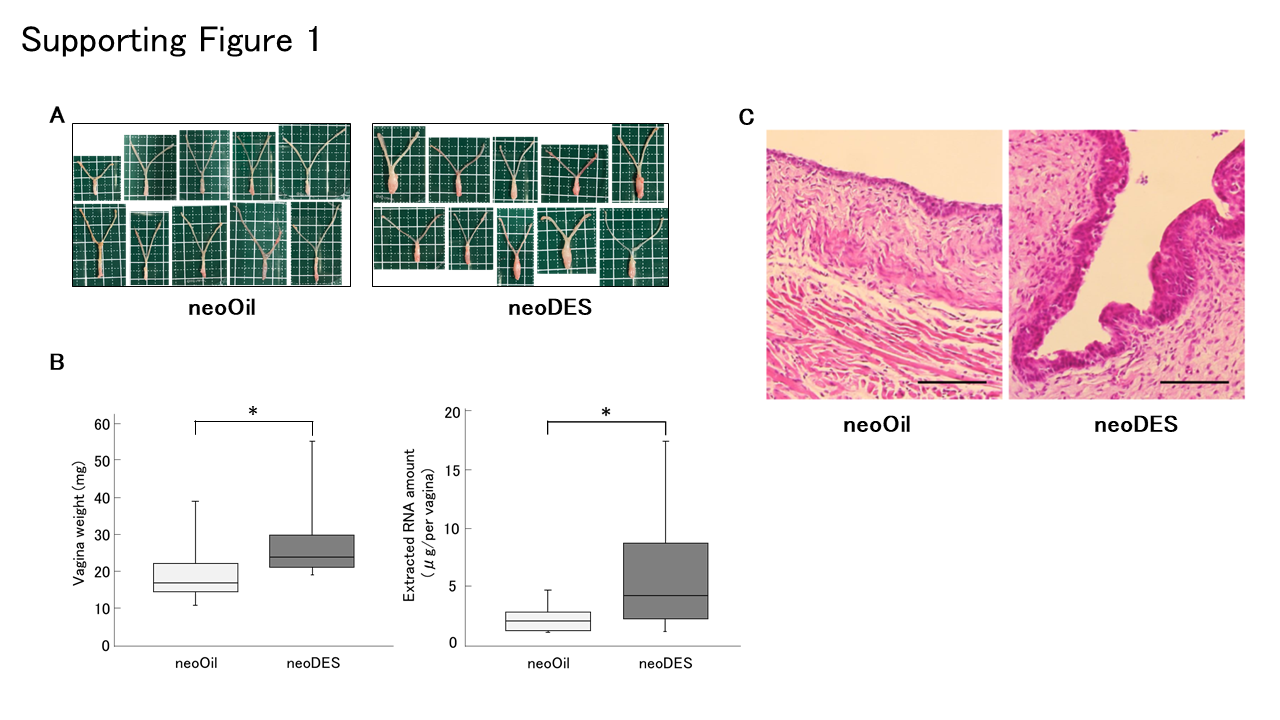

Supplement: S1 Fig — (A) Picture of dissected vagina with uterus. The vagina of control group mice (neoOil) and DES-treated (neoDES). (B) (Left) Dissected vagina weight (neoOil vs neoDES). (n = 10; t-test, *p < 0.05). (Right) Extracted RNA amount from intact vagina (neoOil vs neoDES). (n = 3; t-test, *p < 0.05). As the standard deviation of their body weight was much smaller than the vagina weight, these values were directly compared. (C) Histological observation was performed for the vaginas tissue sections of neoOil and neoDES stained by hematoxylin and eosin. Magnification, ×200; scale bar = 100μm. (TIF) [file pone.0280421.s001.tif]

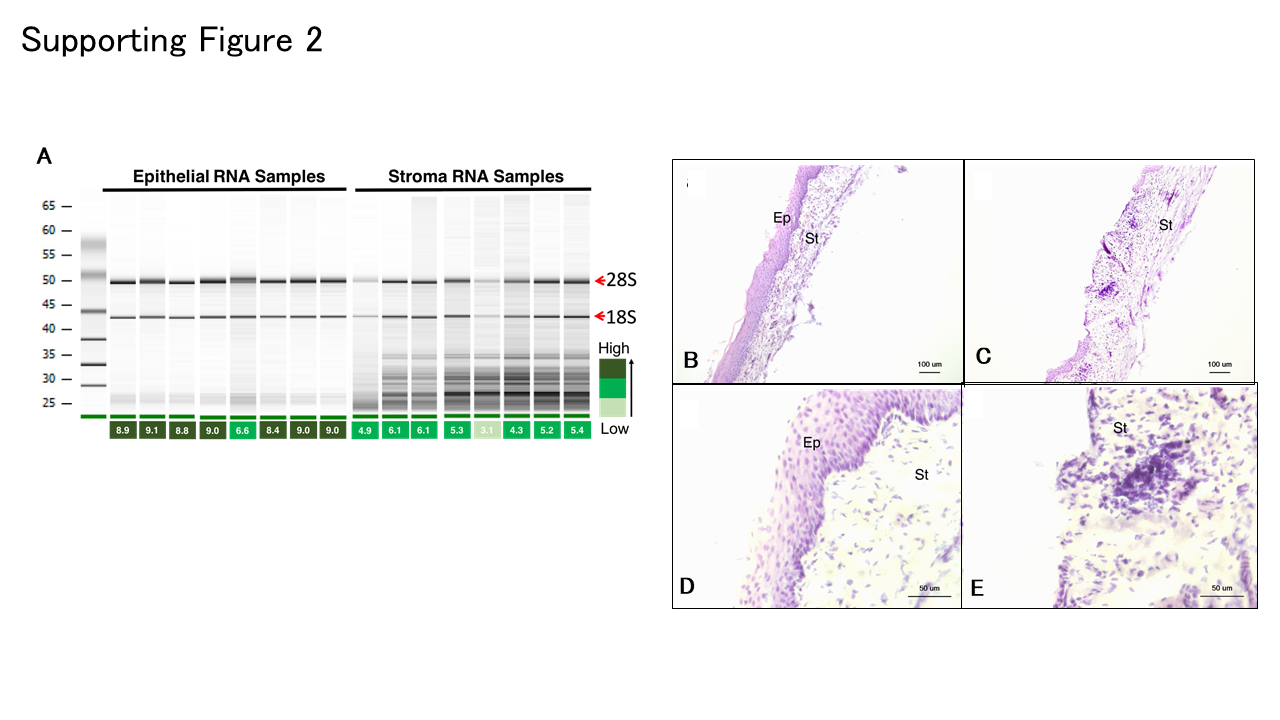

Supplement: S2 Fig — (A) Electrophoresis result and corresponding RNA Integrity Number (RIN) value provided by Bioanalyzer™. Part of the sample results is shown here. The left 8 samples were from epithelial RNA, and the right 8 samples are from stroma RNA. The upper band in the electrophoresis gel picture shows the 28 S rRNA, and the lower band shows 18 S rRNA. The RIN value is calculated by a set of algorithms using the Agilent Bioanalyzer machine. RIN value varies from 1 to 10, with 1 being the most degraded profile and 10 being the most intact. A Higher RIN value is shown in darker green color in this Figure. (B) to (E) Vaginal histology before and after epithelium-stroma separation. Sections were stained with Hematoxylin and Eosin. (TIF) [file pone.0280421.s002.tif]

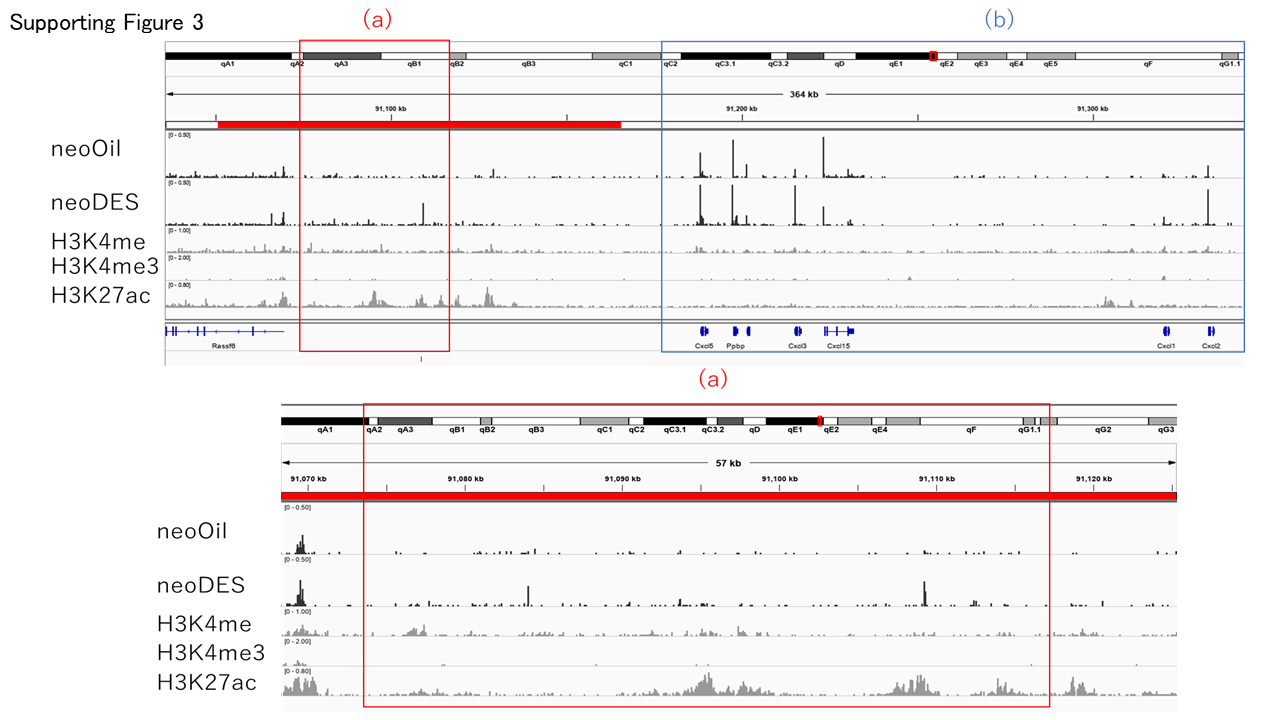

Supplement: S3 Fig — (A) CAGE result showing the relationship of CXC chemokine cluster and peRNAs. CAGE result (Control group and neoDES mice) and public ChIP-seq histone track (Placenta H3K4me, Placenta H3K4me3, and Placenta H3K27ac, showing a comprehensive survey of cis-regulatory elements in the mouse genome by using ChIP-seq to identify transcription factor binding sites and chromatin modification profiles in 8th-week mouse (C57BL/6) placenta.) The region including putative eRNAs (a) is about 100 kb upstream of the CXC chemokine cluster region (b). Magnification of (a) and (b) are shown in Figure. (B) The region including peRNA1 and peRNA2. peRNA1 and peNRA2 were up-regulated in neoDES mice. S1SI_Caption> (TIF) [file pone.0280421.s003.TIF]
