# Supplementary material for: Neonatal administration of synthetic estrogen, diethylstilbestrol to mice up-regulates inflammatory Cxclchemokines located in the 5qE1 region in the vaginal epithelium
Source: PLoS One. 2023 Mar 16;18(3):e0280421. doi: 10.1371/journal.pone.0280421 (PMC10019738; doi:10.1371/journal.pone.0280421)
Supplement: S1 Table — (DOCX) [file pone.0280421.s004.docx]

Supporting Table S1. 361 up-regulated transcripts with coding gene annotation.

| Cluster | LogFC | LogCPM | FDR | Gene |
| --- | --- | --- | --- | --- |
| chr1_121318733_121318862_- | 2.38 | 2.93 | 0.0024 | Inhbb |
| chr1_129915308_129915316_- | 1.58 | 2.98 | 0.029 | Zranb3 |
| chr1_133640594_133640618_+ | 4.69 | -0.47 | 2.20E-05 | Slc26a9 |
| chr1_134195574_134195641_+ | 2.03 | 1.3 | 0.012 | Klhdc8a |
| chr1_166081931_166082023_+ | 2.1 | 2.74 | 0.0037 | F5 |
| chr1_173461795_173461815_- | 7.65 | -0.6 | 1.60E-06 | Itln1 |
| chr1_173464926_173465018_- | 9.98 | 1.59 | 1.20E-11 | Itln1 |
| chr1_173465385_173465453_- | 8.61 | 8.24 | 4.80E-21 | Itln1 |
| chr1_17592823_17592883_+ | 1.95 | 1.07 | 0.035 | Pi15 |
| chr1_186651105_186651158_- | 2.37 | 1.26 | 0.0008 | Mosc2 |
| chr1_189821705_189821745_+ | 6.14 | 0.06 | 3.50E-08 | Esrrg |
| chr1_193007127_193007136_- | 2.5 | 4.48 | 3.00E-05 | Atf3 |
| chr1_193007201_193007205_- | 3.58 | -0.89 | 0.04 | Atf3 |
| chr1_55109639_55109743_+ | 1.66 | 5.16 | 0.0041 | Coq10b |
| chr1_75484052_75484099_- | 2.14 | 0.92 | 0.0071 | Obsl1 |
| chr1_85008975_85009018_+ | 6.66 | 0.54 | 1.50E-09 | Rpl19-ps1 |
| chr1_93355804_93355927_- | 1.48 | 5.68 | 0.012 | Per2 |
| chr1_94728407_94728446_+ | 1.51 | 4.51 | 0.011 | Gpc1 |
| chr10_117066551_117066556_+ | 1.74 | 2.43 | 0.046 | Cpm |
| chr10_21878353_21878411_+ | 5.31 | -0.68 | 2.40E-05 | H60b |
| chr10_24647200_24647392_- | 2.13 | 8.38 | 1.20E-05 | Arg1 |
| chr10_25798311_25798324_- | 3.01 | -1.34 | 0.046 | Tmem200a |
| chr10_42654699_42654811_+ | 2.82 | 0.18 | 0.0068 | Scml4 |
| chr10_44178381_44178384_- | 2.27 | 0.6 | 0.0062 | Prdm1 |
| chr10_44178478_44178586_- | 1.8 | 4.1 | 0.0084 | Prdm1 |
| chr10_5863999_5864079_- | 1.94 | 1.28 | 0.011 | Gm221 |
| chr10_94013695_94013699_+ | 7.58 | -0.65 | 0.00032 | Tmcc3 |
| chr11_100721644_100721649_+ | 5.33 | -0.63 | 0.0069 | Stat5a |
| chr11_103222775_103222891_- | 2.46 | 2.6 | 0.0025 | Arhgap27 |
| chr11_103224947_103225009_- | 2.09 | 1.33 | 0.017 | Arhgap27 |
| chr11_110829221_110829223_+ | 6.36 | -1.66 | 0.023 | Kcnj16 |
| chr11_110829334_110829346_+ | 2.82 | -0.34 | 0.0035 | Kcnj16 |
| chr11_113480639_113480654_+ | 2.82 | 0.07 | 0.0069 | Sstr2 |
| chr11_116099507_116099518_- | 3.06 | -0.99 | 0.024 | Evpl |
| chr11_116100171_116100266_- | 3.6 | 0.3 | 1.70E-05 | Evpl |
| chr11_116393204_116393206_+ | 4.09 | -1.63 | 0.024 | Sphk1 |
| chr11_116611244_116611253_- | 6.32 | -1.68 | 0.0038 | Gm11735 |
| chr11_116613642_116613658_+ | 9.79 | 1.4 | 1.30E-12 | BC018473 |
| chr11_118338138_118338333_+ | 1.3 | 6.53 | 0.018 | Engase |
| chr11_121380987_121380992_- | 4.15 | -1.58 | 0.04 | Zfp750 |
| chr11_121381078_121381151_- | 2 | 3.25 | 0.0049 | Zfp750 |
| chr11_34934571_34934636_+ | 1.19 | 6.46 | 0.043 | Slit3 |
| chr11_4166536_4166538_+ | 6.02 | -0.04 | 5.00E-06 | Lif |
| chr11_48805748_48805767_- | 2.74 | 3.37 | 0.0079 | Gm12185 |
| chr11_48805748_48805767_- | 2.74 | 3.37 | 0.0079 | Tgtp1 |
| chr11_50044178_50044248_+ | 2.03 | 1.24 | 0.0088 | Mgat4b |
| chr11_53164481_53164483_+ | 2.57 | -0.11 | 0.038 | Aff4 |
| chr11_57886436_57886572_- | 6.66 | -1.43 | 0.0013 | Gm12248 |
| chr11_6558502_6558594_+ | 1.79 | 1.53 | 0.023 | Ramp3 |
| chr11_69714458_69714466_- | 2.49 | -0.18 | 0.017 | 2810408A11Rik |
| chr11_69778267_69778312_+ | 1.87 | 2.5 | 0.0073 | Cldn7 |
| chr11_70354104_70354143_+ | 2.56 | 1.1 | 0.042 | Pld2 |
| chr11_75344784_75344786_+ | 6.39 | -1.63 | 0.0028 | Slc43a2 |
| chr11_75813434_75813490_+ | 2.3 | 0.06 | 0.011 | 1700016K19Rik |
| chr11_81928682_81928698_+ | 1.78 | 4.86 | 0.013 | Ccl8 |
| chr11_86399690_86399745_- | 1.81 | 1.23 | 0.034 | Vmp1 |
| chr11_86400291_86400511_- | 1.25 | 5.99 | 0.036 | Vmp1 |
| chr11_9018514_9018631_+ | 1.87 | 2.93 | 0.0089 | Upp1 |
| chr11_95275443_95275445_+ | 3.04 | -1.01 | 0.01 | Spop |
| chr11_95867254_95867259_- | 2.63 | -1.05 | 0.033 | Igf2bp1 |
| chr11_98562653_98562659_+ | 2.39 | 1.23 | 0.0024 | Csf3 |
| chr11_99354418_99354449_- | 2.07 | 5.35 | 0.0017 | Krt23 |
| chr11_99980462_99980486_- | 2.14 | 0.86 | 0.01 | Krt13 |
| chr11_99980636_99980758_- | 2.31 | 2.88 | 0.0005 | Krt13 |
| chr11_99981016_99981092_- | 1.88 | 3.48 | 0.0065 | Krt13 |
| chr11_99981745_99981754_- | 3.43 | 1.36 | 0.00035 | Krt13 |
| chr11_99982431_99982477_- | 1.82 | 1.76 | 0.02 | Krt13 |
| chr11_99982654_99982736_- | 2.11 | 2.84 | 0.00089 | Krt13 |
| chr11_99982836_99982884_- | 2.19 | 9.11 | 1.20E-05 | Krt13 |
| chr12_111517043_111517310_- | 2.1 | 3.81 | 5.20E-05 | Dio3os |
| chr12_111517043_111517310_- | 2.1 | 3.81 | 5.20E-05 | Mir1247 |
| chr12_111517438_111517441_+ | 2.16 | 0.79 | 0.0063 | Dio3 |
| chr12_16746904_16747062_- | 2.31 | 2.37 | 0.00015 | Greb1 |
| chr12_17999635_17999698_+ | 1.71 | 1.3 | 0.05 | Gm4983 |
| chr12_24319597_24319613_- | 3.61 | -0.57 | 0.0012 | Gm4109 |
| chr12_25257445_25257500_+ | 1.73 | 2.38 | 0.029 | Grhl1 |
| chr12_25365709_25365882_- | 1.64 | 4.4 | 0.004 | Cys1 |
| chr12_25366657_25366701_- | 1.95 | 1.34 | 0.0094 | Cys1 |
| chr12_28027546_28027589_- | 2.11 | 1.23 | 0.02 | Sox11 |
| chr12_36719359_36719512_+ | 1.97 | 7.25 | 0.0012 | Agr2 |
| chr12_77634146_77634198_+ | 4.39 | 0.37 | 0.00063 | Plekhg3 |
| chr12_77634294_77634298_+ | 3.4 | -1.48 | 0.049 | Plekhg3 |
| chr12_85262679_85262686_- | 3.45 | -1.02 | 0.0041 | Numb |
| chr12_86814817_86814904_+ | 2.67 | 5.45 | 6.50E-09 | Fos |
| chr12_86815022_86815063_+ | 3.2 | -0.88 | 0.0046 | Fos |
| chr12_86940232_86940436_+ | 2.75 | 6.65 | 2.80E-05 | Jdp2 |
| chr13_106061119_106061122_- | 2.25 | -0.49 | 0.043 | Rnf180 |
| chr13_111185251_111185399_+ | 1.47 | 6.51 | 0.0057 | Plk2 |
| chr13_21902303_21902306_+ | 9.34 | 5.46 | 0.00012 | Hist1h2ao |
| chr13_37918779_37918816_+ | 1.69 | 3.94 | 0.048 | Rreb1 |
| chr13_40955447_40955562_+ | 2.25 | 2.5 | 0.0003 | Gcnt2 |
| chr13_40955575_40955586_+ | 2.74 | -0.41 | 0.0052 | Gcnt2 |
| chr13_4149854_4149991_- | 1.92 | 2.93 | 0.038 | Akr1c18 |
| chr13_51850102_51850165_- | 3.4 | 0.14 | 0.00015 | Sema4d |
| chr13_51851974_51852004_- | 3.03 | 1.45 | 0.002 | Sema4d |
| chr13_51942042_51942058_+ | 1.98 | 6.23 | 6.30E-05 | Gadd45g |
| chr13_55937445_55937557_- | 2.07 | 5.16 | 5.90E-05 | Pitx1 |
| chr13_55937695_55937732_- | 2.75 | 1.22 | 0.00015 | Pitx1 |
| chr13_72766184_72766319_+ | 1.6 | 4.7 | 0.0063 | Irx2 |
| chr13_76091556_76091559_+ | 9.07 | 7.7 | 0.00031 | Gm6311 |
| chr14_19071482_19071488_- | 1.84 | 0.9 | 0.033 | Nr1d2 |
| chr14_41998453_41998455_- | 3.94 | -0.01 | 1.30E-05 | Sftpd |
| chr14_47809042_47809121_- | 1.66 | 1.92 | 0.023 | Gch1 |
| chr14_56332210_56332335_- | 2.84 | 4.62 | 0.033 | Tgm1 |
| chr14_57752417_57752452_- | 3.46 | -1 | 0.0098 | Gjb6 |
| chr14_69273748_69273937_- | 1.53 | 7.16 | 0.018 | Adam28 |
| chr14_76637374_76637383_- | 4 | 1.41 | 6.50E-09 | Rps2-ps6 |
| chr15_100135400_100135427_+ | 2.72 | -0.99 | 0.022 | Mettl7a1 |
| chr15_101097274_101097318_+ | 1.93 | 5.63 | 0.00014 | Nr4a1 |
| chr15_101524300_101524352_- | 2.38 | 1.57 | 0.014 | Krt6a |
| chr15_54109856_54109860_- | 4.07 | -0.53 | 0.0021 | Tnfrsf11b |
| chr15_54402707_54402765_+ | 3.1 | 2.86 | 0.0001 | Mal2 |
| chr15_57817690_57817706_+ | 4.73 | -0.43 | 6.30E-05 | Fam83a |
| chr15_59480135_59480301_+ | 1.46 | 4.91 | 0.033 | Trib1 |
| chr15_74502997_74503002_- | 4.27 | 4.08 | 2.30E-08 | Arc |
| chr15_79177931_79178073_+ | 1.71 | 3.65 | 0.019 | Maff |
| chr15_80807967_80808047_+ | 1.99 | 2.06 | 0.025 | Sgsm3 |
| chr15_84686868_84686977_- | 6.95 | -1.2 | 0.00014 | Phf21b |
| chr15_92227341_92227514_+ | 1.89 | 3.14 | 0.0045 | Pdzrn4 |
| chr15_92227609_92227618_+ | 2.65 | -0.32 | 0.0082 | Pdzrn4 |
| chr16_30064278_30064281_+ | 3.47 | -1.42 | 0.024 | Hes1 |
| chr16_32736324_32736461_+ | 1.52 | 4.84 | 0.034 | Muc4 |
| chr16_33794164_33794166_+ | 4.6 | -0.55 | 0.00049 | Muc13 |
| chr16_33794228_33794232_+ | 4.69 | -1.2 | 0.0017 | Muc13 |
| chr16_36130827_36130919_- | 4.39 | 0.41 | 7.70E-06 | Csta |
| chr16_36131399_36131401_- | 6.06 | -1.86 | 0.012 | Csta |
| chr16_36367794_36367798_- | 3.14 | -1.25 | 0.013 | BC100530 |
| chr16_36808147_36808264_- | 2.67 | 2.1 | 7.40E-06 | Eaf2 |
| chr16_76000969_76001039_+ | 4.21 | 0.87 | 4.30E-06 | Gm15555 |
| chr16_84773896_84773899_+ | 2.99 | -1.05 | 0.012 | Jam2 |
| chr17_23814391_23814481_- | 2.16 | 6.3 | 0.0021 | Tnfrsf12a |
| chr17_23990723_23990758_+ | 3.67 | 3.83 | 4.00E-11 | Prss32 |
| chr17_24175114_24175244_+ | 2.7 | 5.3 | 7.70E-06 | Prss27 |
| chr17_24987038_24987060_- | 2.43 | -0.24 | 0.014 | Fahd1 |
| chr17_26645381_26645424_- | 1.79 | 6.55 | 0.00019 | Dusp1 |
| chr17_27865817_27865928_- | 1.72 | 4.69 | 0.014 | Spdef |
| chr17_29505903_29505920_+ | 2.75 | -0.39 | 0.0066 | Fgd2 |
| chr17_29627687_29627851_+ | 1.36 | 5.7 | 0.036 | Pim1 |
| chr17_31432729_31432793_+ | 4.07 | 0.34 | 4.10E-06 | Slc37a1 |
| chr17_31433130_31433214_+ | 2.46 | 3.1 | 0.0043 | Slc37a1 |
| chr17_31433648_31433757_+ | 1.88 | 4.03 | 0.04 | Slc37a1 |
| chr17_35137290_35137308_- | 9.43 | 5.01 | 9.00E-05 | D17H6S56E-5 |
| chr17_35338948_35338953_- | 2.29 | 1.8 | 0.00063 | Tnf |
| chr17_35763494_35763598_- | 1.68 | 5.69 | 0.005 | Gm9573 |
| chr17_35958627_35958638_+ | 2.37 | 6.94 | 9.00E-07 | Ier3 |
| chr17_36131415_36131489_+ | 2.13 | 4.44 | 0.00019 | A930015D03Rik |
| chr17_36131415_36131489_+ | 2.13 | 4.44 | 0.00019 | RP24-443D19.4 |
| chr17_36179940_36179942_+ | 5.37 | -0.62 | 1.30E-05 | Gm6034 |
| chr17_36220442_36220477_- | 7.6 | -0.66 | 2.40E-05 | H2-Bl |
| chr17_36248596_36248640_- | 6.03 | 4.59 | 1.70E-24 | C920025E04Rik |
| chr17_36258361_36258411_- | 2.02 | 1.74 | 0.003 | H2-T10 |
| chr17_37620479_37620525_- | 1.99 | 3.06 | 0.00053 | H2-M2 |
| chr17_44872814_44872816_+ | 5.62 | -2.14 | 0.049 | Gm10497 |
| chr17_47971332_47971538_- | 1.71 | 4.26 | 0.0069 | Mdfi |
| chr17_7152666_7152772_- | 2.22 | 4.14 | 0.0015 | Rsph3b |
| chr17_7152973_7153094_- | 1.51 | 4.81 | 0.021 | Rsph3b |
| chr18_15562150_15562161_- | 2.81 | 0.78 | 0.00081 | Aqp4 |
| chr18_20217762_20217878_- | 1.2 | 6.32 | 0.044 | Dsc2 |
| chr18_20217901_20217903_- | 2.24 | -0.06 | 0.035 | Dsc2 |
| chr18_20217925_20217930_- | 2.52 | 0.52 | 0.038 | Dsc2 |
| chr18_20218003_20218018_- | 2.18 | 5.36 | 0.0087 | Dsc2 |
| chr18_35020852_35020875_+ | 2.75 | 8.15 | 6.80E-09 | Egr1 |
| chr18_35020908_35020912_+ | 4.5 | -1.33 | 0.0029 | Egr1 |
| chr18_35021004_35021007_+ | 4.61 | -1.24 | 0.0017 | Egr1 |
| chr18_36675422_36675479_- | 2.96 | 5.83 | 2.30E-09 | Hbegf |
| chr18_36720554_36720556_+ | 2.63 | -1.3 | 0.042 | Ankhd1 |
| chr18_37901715_37901748_+ | 8.9 | 0.54 | 4.50E-09 | Pcdhgc5 |
| chr18_60535660_60535690_+ | 2.81 | 3.56 | 0.0027 | Iigp1 |
| chr18_61173742_61173893_- | 2.17 | 1.35 | 0.0037 | Slc6a7 |
| chr18_61419640_61419643_- | 6.7 | 5.36 | 1.20E-34 | Rps2-ps10 |
| chr18_62070979_62071002_- | 3.3 | -1.14 | 0.0084 | Ablim3 |
| chr18_62071192_62071263_- | 2.68 | 4.22 | 3.50E-06 | Ablim3 |
| chr18_65210576_65210729_+ | 1.71 | 3.39 | 0.0096 | Nedd4l |
| chr18_66618182_66618259_+ | 1.98 | 4.32 | 0.00092 | Pmaip1 |
| chr18_82861617_82861689_+ | 2.18 | 0.77 | 0.015 | Gm10524 |
| chr19_11124908_11125033_- | 1.42 | 4.65 | 0.027 | AW112010 |
| chr19_18824186_18824346_+ | 1.72 | 4.17 | 0.019 | Trpm6 |
| chr19_20452770_20452779_- | 3.56 | -1.37 | 0.033 | Anxa1 |
| chr19_20457384_20457505_- | 1.47 | 4.4 | 0.035 | Anxa1 |
| chr19_20465077_20465220_- | 1.48 | 9.99 | 0.0044 | Anxa1 |
| chr19_24248490_24248684_- | 1.61 | 5.18 | 0.0085 | Tjp2 |
| chr19_34082613_34082617_+ | 4.44 | -1.37 | 0.0035 | Lipk |
| chr19_34082650_34082824_+ | 3.66 | 5.84 | 2.00E-16 | Lipk |
| chr19_34141835_34142019_+ | 2.92 | 2.19 | 0.0023 | Lipn |
| chr19_37412745_37412863_- | 2.49 | 0.21 | 0.0031 | Ide |
| chr19_47582366_47582432_- | 1.78 | 2.12 | 0.038 | Obfc1 |
| chr19_47582609_47582612_- | 6.34 | -1.67 | 0.0084 | Obfc1 |
| chr19_47928596_47928618_+ | 2.78 | 0.44 | 0.00069 | Gsto1 |
| chr19_47929459_47929570_+ | 1.64 | 10.33 | 0.0016 | Gsto1 |
| chr19_53603582_53603637_+ | 3.2 | 4.7 | 5.80E-06 | Dusp5 |
| chr19_53867756_53867855_+ | 3.59 | 0.13 | 7.40E-05 | Rbm20 |
| chr19_7369705_7369715_- | 2.2 | -0.3 | 0.038 | Mark2 |
| chr2_104689593_104689672_- | 1.64 | 2.88 | 0.027 | Prrg4 |
| chr2_109533720_109533778_+ | 1.92 | 2.92 | 0.0019 | Bdnf |
| chr2_112079598_112079608_+ | 2.83 | -0.51 | 0.0062 | Lpcat4 |
| chr2_112082762_112082764_+ | 2.79 | -0.7 | 0.044 | Lpcat4 |
| chr2_119000220_119000308_+ | 1.73 | 2.89 | 0.013 | Gm14137 |
| chr2_127555597_127555703_- | 1.38 | 6.7 | 0.028 | Mall |
| chr2_129024443_129024541_+ | 1.84 | 4.43 | 0.003 | Slc20a1 |
| chr2_131317585_131317702_+ | 1.39 | 5.96 | 0.017 | Smox |
| chr2_137702594_137702627_- | 4.89 | 0.2 | 1.30E-06 | Gm14062 |
| chr2_151798613_151798627_- | 2.03 | 0.76 | 0.036 | Fam110a |
| chr2_165687515_165687524_- | 2.46 | 0.27 | 0.0068 | Zmynd8 |
| chr2_167174616_167174745_- | 1.43 | 5.57 | 0.048 | B4galt5 |
| chr2_176991111_176991188_- | 9.56 | 1.18 | 1.60E-10 | Gm14410 |
| chr2_22149115_22149183_+ | 4.34 | 0.6 | 1.00E-06 | Myo3a |
| chr2_24192238_24192240_+ | 5.96 | -0.1 | 2.60E-07 | Il1rn |
| chr2_24192341_24192459_+ | 2.32 | 5.63 | 0.039 | Il1rn |
| chr2_32306894_32306982_- | 1.48 | 3.7 | 0.034 | Slc25a25 |
| chr2_32485752_32485755_+ | 3.15 | -0.46 | 0.042 | Ak1 |
| chr2_39983316_39983448_- | 4.58 | 3.2 | 0.026 | Gm13453 |
| chr2_54092919_54092921_+ | 9 | 0.64 | 1.20E-11 | Gm13505 |
| chr2_90757722_90757727_+ | 3.06 | -1 | 0.043 | Gm17661 |
| chr3_102905991_102906026_+ | 2.24 | 1.15 | 0.016 | Dennd2c |
| chr3_103636719_103636795_+ | 2.05 | 1.26 | 0.0069 | Bcl2l15 |
| chr3_129883754_129883811_+ | 2.94 | 0.95 | 0.0012 | Col25a1 |
| chr3_145312871_145312998_- | 1.79 | 6.59 | 0.0011 | Cyr61 |
| chr3_28664261_28664322_- | 10.48 | 2.08 | 2.30E-20 | Gm6505 |
| chr3_30995621_30995624_+ | 1.41 | 2.87 | 0.049 | Skil |
| chr3_65332299_65332416_+ | 1.6 | 6.29 | 0.023 | Tiparp |
| chr3_69663781_69663922_- | 1.8 | 6.86 | 0.0003 | 1110032A04Rik |
| chr3_83570186_83570381_+ | 1.2 | 7.69 | 0.038 | Sfrp2 |
| chr3_83570718_83570722_+ | 2.37 | -0.42 | 0.023 | Sfrp2 |
| chr3_83594074_83594093_- | 2.98 | -0.38 | 0.012 | Gm16790 |
| chr3_85691162_85691175_- | 3.91 | -0.3 | 0.0096 | Arfip1 |
| chr3_85691162_85691175_- | 3.91 | -0.3 | 0.0096 | Glt28d2 |
| chr3_89876566_89876581_+ | 1.64 | 3.59 | 0.038 | Tpm3 |
| chr3_90341943_90341949_+ | 2.45 | -0.33 | 0.046 | S100a16 |
| chr3_90342388_90342405_+ | 2.41 | 0.16 | 0.04 | S100a16 |
| chr3_92261473_92261492_- | 3.98 | 1.72 | 0.00012 | Sprr3 |
| chr3_95686088_95686181_- | 2.08 | 2.12 | 0.003 | Gm129 |
| chr4_111904745_111904884_+ | 3.89 | 3.91 | 0.044 | Skint3 |
| chr4_111904893_111904898_+ | 6.06 | -1.86 | 0.01 | Skint3 |
| chr4_116806543_116806636_- | 2.52 | 5.81 | 5.80E-06 | Plk3 |
| chr4_118781345_118781486_+ | 1.36 | 7.59 | 0.039 | Slc2a1 |
| chr4_119833962_119834031_+ | 1.55 | 4.06 | 0.012 | Edn2 |
| chr4_128735702_128735712_+ | 4.77 | 1.18 | 2.40E-08 | Rnf19b |
| chr4_132807262_132807331_+ | 2.15 | 0.59 | 0.017 | Map3k6 |
| chr4_137220635_137220694_+ | 2.85 | 0.74 | 0.00077 | Rap1gap |
| chr4_137923834_137923949_- | 2.59 | 1.96 | 0.00014 | Cda |
| chr4_140330099_140330184_- | 2.68 | 6.7 | 5.00E-05 | Padi4 |
| chr4_140401581_140401769_- | 2.07 | 7.81 | 0.00077 | Padi1 |
| chr4_140857099_140857207_+ | 1.62 | 5.25 | 0.0028 | Epha2 |
| chr4_149943318_149943383_+ | 1.56 | 3.6 | 0.012 | Rere |
| chr4_150418632_150418796_- | 1.26 | 6.43 | 0.043 | Per3 |
| chr4_46356490_46356565_+ | 2.69 | 2.29 | 0.0076 | Foxe1 |
| chr4_55545333_55545432_- | 1.54 | 7.53 | 0.035 | Klf4 |
| chr4_62095627_62095653_- | 4.96 | -0.97 | 0.00024 | Rnf183 |
| chr5_113709568_113709648_- | 2.76 | 0.13 | 0.002 | Sgsm1 |
| chr5_115373352_115373405_+ | 2.3 | 3.42 | 0.0052 | Oasl1 |
| chr5_119011115_119011133_+ | 2.12 | -0.08 | 0.043 | Med13l |
| chr5_120120849_120120853_- | 4.1 | -1.63 | 0.021 | Gm2301 |
| chr5_124134474_124134476_- | 6.13 | -1.81 | 0.023 | Clip1 |
| chr5_135422310_135422313_- | 2.51 | -0.45 | 0.026 | Cldn4 |
| chr5_135422555_135422578_- | 2.96 | -1.06 | 0.025 | Cldn4 |
| chr5_135422752_135422841_- | 1.97 | 8.1 | 0.00038 | Cldn4 |
| chr5_137548137_137548144_- | 1.81 | 2.19 | 0.014 | Serpine1 |
| chr5_30764253_30764309_- | 3.29 | 0.2 | 0.00015 | Otof |
| chr5_32438170_32438173_+ | 2.32 | 0.71 | 0.005 | Fosl2 |
| chr5_34064952_34064955_+ | 3.21 | -0.6 | 0.013 | Fgfr3 |
| chr5_35384178_35384245_+ | 2.33 | 1.01 | 0.0023 | Hgfac |
| chr5_36740706_36740827_- | 1.27 | 5.27 | 0.047 | Sorcs2 |
| chr5_3927878_3927917_+ | 3.49 | -1.42 | 0.036 | Akap9 |
| chr5_73797321_73797331_+ | 3.07 | 1.54 | 0.013 | Cwh43 |
| chr5_77543892_77543982_- | 2.06 | 7.3 | 4.00E-06 | Hopx |
| chr5_86947563_86947732_- | 3.27 | 6.17 | 0.0065 | Tmprss11g |
| chr5_87105296_87105441_- | 2.26 | 7.48 | 0.037 | Tmprss11bnl |
| chr5_87105489_87105503_- | 2.8 | -0.2 | 0.0075 | Tmprss11bnl |
| chr5_91188320_91188388_+ | 2.65 | 5.67 | 0.00069 | Cxcl5 |
| chr5_91188497_91188554_+ | 1.75 | 2.62 | 0.015 | Cxcl5 |
| chr5_91197507_91197569_+ | 4.35 | 6.75 | 0.0098 | Ppbp |
| chr5_91197707_91197709_+ | 4.66 | -1.21 | 0.0018 | Ppbp |
| chr5_91198477_91198479_+ | 4.36 | -0.74 | 0.00029 | Ppbp |
| chr5_91198531_91198533_+ | 7.01 | -1.15 | 0.00014 | Ppbp |
| chr5_91215122_91215247_+ | 2.92 | 4.05 | 0.00035 | Cxcl3 |
| chr5_91320265_91320271_+ | 1.88 | 0.8 | 0.032 | Cxcl1 |
| chr5_91332894_91332902_+ | 3.11 | 2.28 | 4.70E-07 | Cxcl2 |
| chr5_92391465_92391468_+ | 2.88 | 1.12 | 0.00021 | Thap6 |
| chr6_114408513_114408525_+ | 3.37 | -1.09 | 0.005 | Hrh1 |
| chr6_117887337_117887357_- | 1.57 | 6.17 | 0.0043 | Fxyd4 |
| chr6_122645989_122645992_+ | 4.58 | 5.84 | 5.10E-21 | Gm10420 |
| chr6_128924925_128925063_- | 1.45 | 4.66 | 0.033 | Gm15987 |
| chr6_135312918_135313092_+ | 1.32 | 7.41 | 0.031 | Emp1 |
| chr6_137519252_137519401_- | 1.56 | 2.27 | 0.044 | Eps8 |
| chr6_137596784_137596806_- | 2.78 | -0.95 | 0.016 | Eps8 |
| chr6_144996026_144996176_- | 6.68 | 5.59 | 2.60E-12 | Bcat1 |
| chr6_145024462_145024619_- | 4.28 | 4.47 | 5.90E-09 | Bcat1 |
| chr6_145024647_145024653_- | 6.15 | -1.8 | 0.0095 | Bcat1 |
| chr6_145024666_145024675_- | 6.23 | -1.74 | 0.0079 | Bcat1 |
| chr6_145813205_145813262_- | 3 | -0.36 | 0.0021 | Bhlhe41 |
| chr6_145813872_145813875_- | 3.86 | -1.15 | 0.011 | Bhlhe41 |
| chr6_145813895_145814111_- | 2.34 | 5.72 | 2.70E-06 | Bhlhe41 |
| chr6_18120746_18120750_+ | 2.9 | -0.29 | 0.0069 | Cftr |
| chr6_50332821_50332931_- | 1.51 | 3.53 | 0.02 | Osbpl3 |
| chr6_53989519_53989715_+ | 1.9 | 3.72 | 0.00053 | Chn2 |
| chr6_65540377_65540421_+ | 3.7 | 2.71 | 6.20E-08 | Tnip3 |
| chr6_66987331_66987423_- | 2.22 | 5.91 | 0.00076 | Gadd45a |
| chr6_86619105_86619231_- | 2.01 | 6.98 | 0.012 | Mxd1 |
| chr6_97437753_97437955_- | 2.26 | 3.78 | 0.011 | Frmd4b |
| chr7_106844276_106844381_- | 1.43 | 2.78 | 0.044 | Slco2b1 |
| chr7_107413159_107413172_+ | 8.32 | -0.01 | 3.60E-07 | Gpx2-ps1 |
| chr7_107433933_107434093_+ | 1.94 | 3.43 | 0.0015 | P4ha3 |
| chr7_108709378_108709381_- | 3.42 | 2.4 | 0.023 | Art2a-ps |
| chr7_113584577_113584607_- | 3 | 1.27 | 4.30E-05 | Gm4759 |
| chr7_116073664_116073693_- | 1.82 | 4.63 | 0.0037 | Nlrp10 |
| chr7_121862033_121862042_+ | 5.07 | -0.13 | 1.60E-06 | Calcb |
| chr7_121862131_121862135_+ | 3.45 | -1.01 | 0.0087 | Calcb |
| chr7_121862154_121862171_+ | 2.69 | 4.5 | 0.023 | Calcb |
| chr7_123381131_123381331_- | 1.71 | 3.17 | 0.0062 | Plekha7 |
| chr7_126602746_126602821_- | 4.58 | 4.09 | 5.90E-15 | Gp2 |
| chr7_147178379_147178462_- | 1.39 | 5.79 | 0.015 | Adam8 |
| chr7_149024972_149025007_+ | 1.98 | 9.43 | 0.00014 | Muc5b |
| chr7_19895358_19895395_- | 1.84 | 3.61 | 0.012 | Fosb |
| chr7_20382566_20382573_- | 7.21 | -0.99 | 0.00011 | Cblc |
| chr7_20408018_20408158_- | 1.37 | 5.69 | 0.036 | Bcl3 |
| chr7_25561301_25561303_+ | 1.83 | 5.25 | 0.048 | Ceacam10 |
| chr7_26472148_26472156_+ | 2.29 | -0.13 | 0.036 | Tgfb1 |
| chr7_26601864_26601885_- | 5.31 | -0.68 | 0.00072 | Cyp2s1 |
| chr7_29747422_29747439_- | 2.32 | 1.2 | 0.0035 | Actn4 |
| chr7_30978817_30978834_- | 4.56 | 0.24 | 3.10E-07 | Capns1 |
| chr7_35597673_35597736_- | 3.12 | 2.34 | 0.0049 | Chst8 |
| chr7_4745106_4745203_+ | 1.87 | 2.19 | 0.0094 | Rpl28 |
| chr7_4923445_4923482_- | 3.59 | 0.47 | 0.00013 | Gm1078 |
| chr7_52960785_52960787_+ | 2.12 | 0.64 | 0.023 | Dbp |
| chr7_52970911_52970915_+ | 4.02 | -1.68 | 0.032 | Rpl18 |
| chr7_54388070_54388112_- | 3.28 | -0.83 | 0.0075 | Ptpn5 |
| chr7_89137152_89137311_- | 1.9 | 3.6 | 0.017 | Bnc1 |
| chr7_96666805_96666831_- | 2.81 | -1.18 | 0.028 | Prss23 |
| chr8_107812934_107813002_- | 3.35 | 0.38 | 5.30E-05 | 4931428F04Rik |
| chr8_109128724_109128801_+ | 2.06 | 0.27 | 0.026 | Cdh1 |
| chr8_109394686_109394769_+ | 1.65 | 4.12 | 0.048 | Has3 |
| chr8_109963460_109963488_- | 5.67 | 8.37 | 2.80E-36 | Rps26-ps1 |
| chr8_116372634_116372664_- | 3.73 | 0.42 | 6.00E-06 | Adamts18 |
| chr8_122637995_122638098_+ | 1.59 | 5.93 | 0.042 | 6430548M08Rik |
| chr8_15119327_15119361_+ | 6.08 | -1.85 | 0.026 | Myom2 |
| chr8_20020277_20020454_- | 1.85 | 6.12 | 0.00035 | 2610005L07Rik |
| chr8_3584249_3584252_- | 4.87 | 1.27 | 0.0073 | Gm10186 |
| chr8_35870345_35870398_+ | 2.31 | 4.27 | 0.00041 | Dusp4 |
| chr8_35870664_35870667_+ | 5.62 | -2.14 | 0.049 | Dusp4 |
| chr8_87502569_87502630_- | 1.51 | 6.61 | 0.0037 | Junb |
| chr8_95803609_95803659_- | 3.3 | 2.08 | 3.90E-07 | Ces1f |
| chr9_110921747_110921835_+ | 3.33 | 9.9 | 9.70E-16 | Ltf |
| chr9_120448817_120448822_+ | 4.48 | -1.36 | 0.015 | Entpd3 |
| chr9_120448831_120448841_+ | 5.82 | 0.56 | 1.00E-07 | Entpd3 |
| chr9_21936086_21936104_- | 2.03 | 1.29 | 0.008 | Acp5 |
| chr9_49235055_49235163_- | 3.3 | 2.88 | 0.00031 | Ankk1 |
| chr9_50302244_50302248_+ | 3.29 | -0.81 | 0.002 | 1600029D21Rik |
| chr9_50302577_50302662_+ | 1.92 | 6.5 | 4.60E-05 | 1600029D21Rik |
| chr9_55174723_55174785_+ | 1.9 | 1.5 | 0.035 | AI118078 |
| chr9_57982014_57982183_+ | 2.51 | 4.08 | 7.30E-05 | Stra6 |
| chr9_76893463_76893467_- | 7.06 | -1.11 | 9.40E-05 | Tinag |
| chr9_97933509_97933673_- | 1.35 | 3.87 | 0.042 | Clstn2 |
| chrX_137442026_137442179_+ | 2.94 | 2.66 | 0.019 | Vsig1 |
| chrX_18744364_18744370_- | 2.09 | 0.64 | 0.011 | Gm14636 |
| chrX_21292063_21292261_+ | 2.35 | 3.3 | 0.00022 | 5S_rRNA |
| chrX_21292063_21292261_+ | 2.35 | 3.3 | 0.00022 | Slc6a14 |
| chrX_50251786_50251847_+ | 6.88 | 0.75 | 3.00E-11 | Rps2-ps13 |
| chrX_50252132_50252203_+ | 6.92 | -1.25 | 0.04 | Rps2-ps13 |
| chrX_5992816_5992861_+ | 4.63 | 0.31 | 5.20E-07 | Shroom4 |
| chrX_71518020_71518082_+ | 3.16 | 0.73 | 0.0013 | Rpl10 |
| chrX_71518020_71518082_+ | 3.16 | 0.73 | 0.0013 | Snora70 |
| chrX_97970974_97971022_+ | 1.93 | 0.47 | 0.039 | Dlg3 |
