# Supplementary material for: Neonatal administration of synthetic estrogen, diethylstilbestrol to mice up-regulates inflammatory Cxclchemokines located in the 5qE1 region in the vaginal epithelium
Source: PLoS One. 2023 Mar 16;18(3):e0280421. doi: 10.1371/journal.pone.0280421 (PMC10019738; doi:10.1371/journal.pone.0280421)
Supplement: S2 Table — (DOCX) [file pone.0280421.s005.docx]

Supporting Table 2. Up-regulated transcripts from genes related to *Cxcl* chemokine.

| Cluster | LogFC | LogCPM | FDR | Gene |
| --- | --- | --- | --- | --- |
| chr5_86915500_86915651_- | 4.18 | 0.21 | 4.00E-06 | * |
| chr5_86916286_86916290_- | 3.35 | -1.09 | 0.017 | * |
| chr5_86940584_86940588_- | 3.67 | -1.28 | 0.012 | * |
| chr5_86947563_86947732_- | 3.27 | 6.17 | 0.0065 | Tmprss11g |
| chr5_87085426_87085487_- | 4.83 | -1.08 | 0.00055 | * |
| chr5_87087723_87087865_- | 2.59 | 1.72 | 0.04 | * |
| chr5_87089363_87089366_- | 6.24 | -1.74 | 0.0052 | * |
| chr5_87094109_87094120_- | 3.13 | -1.25 | 0.035 | * |
| chr5_87097973_87098010_- | 4.27 | 0.28 | 6.50E-07 | * |
| chr5_87105296_87105441_- | 2.26 | 7.48 | 0.037 | Tmprss11bnl |
| chr5_87105489_87105503_- | 2.8 | -0.2 | 0.0075 | Tmprss11bnl |
| chr5_91093705_91093760_- | 3.36 | 0.08 | 0.00069 | * |
| chr5_91109276_91109308_- | 3.73 | 1.62 | 6.40E-06 | * |
| chr5_91188320_91188388_+ | 2.65 | 5.67 | 0.00069 | *Cxcl*5 |
| chr5_91188497_91188554_+ | 1.75 | 2.62 | 0.015 | *Cxcl*5 |
| chr5_91189143_91189171_- | 3.18 | 0.94 | 1.10E-05 | * |
| chr5_91197507_91197569_+ | 4.35 | 6.75 | 0.0098 | Ppbp |
| chr5_91197707_91197709_+ | 4.66 | -1.21 | 0.0018 | Ppbp |
| chr5_91198477_91198479_+ | 4.36 | -0.74 | 0.00029 | Ppbp |
| chr5_91198531_91198533_+ | 7.01 | -1.15 | 0.00014 | Ppbp |
| chr5_91198623_91198716_+ | 4.63 | 2.14 | 3.60E-07 | * |
| chr5_91215122_91215247_+ | 2.92 | 4.05 | 0.00035 | *Cxcl*3 |
| chr5_91320265_91320271_+ | 1.88 | 0.8 | 0.032 | *Cxcl*1 |
| chr5_91332894_91332902_+ | 3.11 | 2.28 | 4.70E-07 | *Cxcl*2 |
| chr5_91503753_91503862_- | 3.09 | 2.46 | 0.0027 | * |
| chr5_92391465_92391468_+ | 2.88 | 1.12 | 0.00021 | Thap6 |

## Supporting Table 3. Primer List

| Name of primer set | Forward | Reverse | Reference/ PrimerBank ID |
| --- | --- | --- | --- |
| TNF-α | CTGTAGCCCACGTCGTAGC | TTGAGATCCATGCCGTTG | [28] |
| Wnt11 | ATGTGCGGACAACCTCAGCTA | CGCATCAGTTTATTGGCTTGG | [13] |
| SMA | GTCCCAGACATCAGGGAGTAA | TCGGATACTTCAGCGTCAGGA | 6671507a1 |
| CK5 | TCTGCCATCACCCCATCTGT | CCTCCGCCAGAACTGTAGGA | 20911031a1 |
| Fos | CGGGTTTCAACGCCGACTA | TTGGCACTAGAGACGGACAGA | 6753894a1 |
| Sfrp2 | CGTGGGCTCTTCCTCTTCG | ATGTTCTGGTACTCGATGCCG | 6677895a1 |
| Cldn7 | GGCCTGATAGCGAGCACTG | GTGACGCACTCCATCCAGA | 8393144a1 |
| CXCL1 | CTGGGATTCACCTCAAGAACATC | CAGGGTCAAGGCAAGCCTC | 6680109a1 |
| CXCL2 | CCAACCACCAGGCTACAGG | GCGTCACACTCAAGCTCTG | 6677885a1 |
| CXCL3 | CAGCCACACTCCAGCCTA | CACAACAGCCCCTGTAGC | [29] |
| CXCL5 | GTTCCATCTCGCCATTCATGC | GCGGCTATGACTGAGGAAGG | 114842397c1 |
| CXCL7 | CTCAGACCTACATCGTCCTGC | GTGGCTATCACTTCCACATCAG | 12963823a1 |
| peRNA No.1 | ATTTGAGCCACACCAAAGCTG | GGAGTGCATTGCAACCCAAG | N/A |
| peRNA No.2 | TGGCTTTGGGCACTGCATAC | CTAGATGGAAGCCCGGTTCTC | N/A |
| peRNA No.3 | TCCTGATTAGGCAGTGTGGC | TGGATGTTGTCAGGACGGTG | N/A |
| peRNA No.4 | GCCCTGCACTTTCAGCATTAG | ATCGAAAGGCAGGCTCACTC | N/A |
| peRNA No.5 | AGCAGCCCTATTTTAGGCGG | GTTGCCAATCCATTCGCCC | N/A |
| peRNA No.6 | GTAGACGTTGAGAGCACCCG | TGCAGTCTCAGCTACATGGG | N/A |
| peRNA No.7 | TAGCTGACTGTGACCATCCAC | CGCTGACACCATTGCATACAC | N/A |
| peRNA No.8 | ACCTGTCAGTAACCCGAGGA | CCTTGAATGTGTGTCACTGCG | N/A |
| peRNA No.9 | CCCTAGCTGCCGTAAGGAAC | GCCATTTACCAGCCGCTATG | N/A |
| peRNA No.10 | TCATGCTACGATACTGGCACC | ACTTTTGCATCTCTCCTCCCG | N/A |
| peRNA No.11 | CCTGGCAGAAGAGTTGACACA | TGAGGCAGCATTTGTTGTGC | N/A |
| peRNA No.12 | GTGCAGACCTGGAGTTGTCA | AGTTTGGTCTGTGTGACTCCC | N/A |
| Spt5 | GGTCCTACTGAGCATTGATGGTGAG | TCAGGCTTCCAGGAGCTTCCCTAGG | [30] |
