# Supplementary material for: Neonatal administration of synthetic estrogen, diethylstilbestrol to mice up-regulates inflammatory Cxclchemokines located in the 5qE1 region in the vaginal epithelium
Source: PLoS One. 2023 Mar 16;18(3):e0280421. doi: 10.1371/journal.pone.0280421 (PMC10019738; doi:10.1371/journal.pone.0280421)
Supplement: S3 Table — (DOCX) [file pone.0280421.s006.docx]

## Supporting Table 3. Primer List

| Name of primer set | Forward | Reverse | Reference/ PrimerBank ID |
| --- | --- | --- | --- |
| TNF-α | CTGTAGCCCACGTCGTAGC | TTGAGATCCATGCCGTTG | [28] |
| Wnt11 | ATGTGCGGACAACCTCAGCTA | CGCATCAGTTTATTGGCTTGG | [13] |
| SMA | GTCCCAGACATCAGGGAGTAA | TCGGATACTTCAGCGTCAGGA | 6671507a1 |
| CK5 | TCTGCCATCACCCCATCTGT | CCTCCGCCAGAACTGTAGGA | 20911031a1 |
| Fos | CGGGTTTCAACGCCGACTA | TTGGCACTAGAGACGGACAGA | 6753894a1 |
| Sfrp2 | CGTGGGCTCTTCCTCTTCG | ATGTTCTGGTACTCGATGCCG | 6677895a1 |
| Cldn7 | GGCCTGATAGCGAGCACTG | GTGACGCACTCCATCCAGA | 8393144a1 |
| CXCL1 | CTGGGATTCACCTCAAGAACATC | CAGGGTCAAGGCAAGCCTC | 6680109a1 |
| CXCL2 | CCAACCACCAGGCTACAGG | GCGTCACACTCAAGCTCTG | 6677885a1 |
| CXCL3 | CAGCCACACTCCAGCCTA | CACAACAGCCCCTGTAGC | [29] |
| CXCL5 | GTTCCATCTCGCCATTCATGC | GCGGCTATGACTGAGGAAGG | 114842397c1 |
| CXCL7 | CTCAGACCTACATCGTCCTGC | GTGGCTATCACTTCCACATCAG | 12963823a1 |
| peRNA No.1 | ATTTGAGCCACACCAAAGCTG | GGAGTGCATTGCAACCCAAG | N/A |
| peRNA No.2 | TGGCTTTGGGCACTGCATAC | CTAGATGGAAGCCCGGTTCTC | N/A |
| peRNA No.3 | TCCTGATTAGGCAGTGTGGC | TGGATGTTGTCAGGACGGTG | N/A |
| peRNA No.4 | GCCCTGCACTTTCAGCATTAG | ATCGAAAGGCAGGCTCACTC | N/A |
| peRNA No.5 | AGCAGCCCTATTTTAGGCGG | GTTGCCAATCCATTCGCCC | N/A |
| peRNA No.6 | GTAGACGTTGAGAGCACCCG | TGCAGTCTCAGCTACATGGG | N/A |
| peRNA No.7 | TAGCTGACTGTGACCATCCAC | CGCTGACACCATTGCATACAC | N/A |
| peRNA No.8 | ACCTGTCAGTAACCCGAGGA | CCTTGAATGTGTGTCACTGCG | N/A |
| peRNA No.9 | CCCTAGCTGCCGTAAGGAAC | GCCATTTACCAGCCGCTATG | N/A |
| peRNA No.10 | TCATGCTACGATACTGGCACC | ACTTTTGCATCTCTCCTCCCG | N/A |
| peRNA No.11 | CCTGGCAGAAGAGTTGACACA | TGAGGCAGCATTTGTTGTGC | N/A |
| peRNA No.12 | GTGCAGACCTGGAGTTGTCA | AGTTTGGTCTGTGTGACTCCC | N/A |
| Spt5 | GGTCCTACTGAGCATTGATGGTGAG | TCAGGCTTCCAGGAGCTTCCCTAGG | [30] |
